# Remote Magnetomechanical Neuromodulation Uncovers Therapeutic Mechanisms for Alleviating Parkinsonian Symptoms in Freely Moving Mice

**DOI:** 10.1002/advs.75097

**Published:** 2026-04-03

**Authors:** Anouk Wolters, Lorenzo Signorelli, Christian Herff, Sophia Gimple, Renzo Riemens, Gunter Kenis, Kim Rijkers, Hamed Shabani, Jyh‐Jang Sun, Yasin Temel, Hans Clusmann, Danijela Gregurec, Sarah‐Anna Hescham

**Affiliations:** ^1^ Department of Neurosurgery Mental Health and Neuroscience Research Institute Maastricht University Maastricht The Netherlands; ^2^ European Graduate School of Neuroscience (EURON) Maastricht University Maastricht The Netherlands; ^3^ Biointerfaces lab, Department of Chemistry and Pharmacy FAU Erlangen‐Nuremberg Erlangen Germany; ^4^ Department of Psychiatry and Neuropsychology Mental Health and Neuroscience Research Institute (MHeNs) Maastricht University Medical Centre Maastricht The Netherlands; ^5^ Academic Center For Epileptology Maastricht/Heeze The Netherlands; ^6^ Centre For Integrative Neuroscience (CIN) Maastricht University Maastricht The Netherlands; ^7^ ATLAS Neuroengineering Leuven Belgium; ^8^ College of Semiconductor Technology and Department of Bioscience Technology Chung Yuan Christian University Taoyuan Taiwan; ^9^ Research Group Experimental Oto‐rhino‐laryngology Department of Neurosciences KU Leuven Leuven Belgium; ^10^ Department of Neurosurgery RWTH Aachen University Hospital Aachen Germany

**Keywords:** DBS, magnetic nanodiscs, magnetomechanical neuromodulation, nanotechnology, Piezo1, TRPV4

## Abstract

To overcome the limitations of invasive neuromodulation systems, we introduce a wireless magnetomechanical approach for remote, minimally‐invasive deep brain stimulation (DBS) without chronically implanted hardware. This method leverages biocompatible magnetite nanodiscs (MNDs) with ground vortex magnetization, which undergo in‐plane transitions under low‐frequency alternating magnetic fields, generating localized piconewton‐scale torques. These torques engage endogenous mechanosensory pathways to modulate neural activity, enabling reversible stimulation without genetic modifications. Calcium‐imaging validated rapid neuromodulatory effects of MNDs in vitro and ex vivo, motivating the application of magnetomechanical DBS to the subthalamic nucleus in mice. We demonstrated remote control of motor behavior in wild‐type mice and significant restoration of motor function in a severe hemiparkinsonian model. Demonstrating efficacy at multiple experimental scales, this work establishes a clinically compatible, electrode‐free neuromodulation technology combining nanoscale engineering with mechanosensory signaling, paving the way toward a minimally‐invasive DBS approach suitable for outpatient use and for patients ineligible for conventional DBS.

## Graphical abstract

Magnetomechanical neuromodulation using magnetic nanodiscs enables remote activation of neurons. In a hemiparkinsonian mouse model, alternating magnetic fields actuate the nanodiscs to generate torque that opens mechanosensitive ion channels within the subthalamic nucleus, thereby modulating basal ganglia motor circuitry. This minimally invasive strategy restores motor function, highlighting a promising approach for precise, wireless neuromodulation of deep brain circuits.

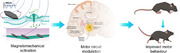

## 1 Introduction

Deep brain stimulation (DBS) is a clinically established, yet invasive, neuromodulation technique requiring surgical implantation of electrodes to deliver electrical stimulation to targeted deep brain regions. It has demonstrated significant therapeutic efficacy in neurological disorders, such as Parkinson's disease (PD), essential tremor, and dystonia. Despite its clinical success, core DBS technology has remained largely unchanged since its initial implementation [1, 2]. Reliance on permanently implanted hardware poses challenges related to surgical complexity, device longevity, and patient acceptance [3]. Moreover, conventional DBS often lacks the spatial precision required to selectively target specific neural circuits because standard electrodes activate large tissue volumes. This can result in off‐target effects, including unwanted motor or cognitive changes, and contribute to variability in clinical outcomes [4]. Clinically, these complications often translate into recurrent hospitalization, hardware infection, or surgical revision, especially in older PD patients or those with comorbidities. These limitations motivate efforts to develop alternative neuromodulation strategies that preserve DBS efficacy while reducing invasiveness [5, 6].

Magnetic nanomaterials, which transduce magnetic fields into biological effects, are emerging as promising tools in bioelectronics and neuromodulation [7]. Their ability to enable remote, localized stimulation without physical wiring makes them attractive for minimally invasive approaches. Magnetoelectric nanoparticles, for example, convert magnetic field input into electrical signals [8, 9], but often contain non‐biocompatible elements that present a risk of long‐term degradation and pose challenges in achieving consistent synthesis and control over physical properties [10, 11]. Magnetothermal approaches use magnetic nanoparticles to dissipate heat under an alternating magnetic field (AMF), thereby activating thermosensitive ion channels [12, 13]. While effective, this approach offers limited temporal precision, requires high concentrations of nanoparticles, raises concerns about the long‐term effects of repeated neuronal heating, and typically relies on genetic modification to sensitize target neurons to thermal stimuli [14, 15].

**FIGURE 1:**
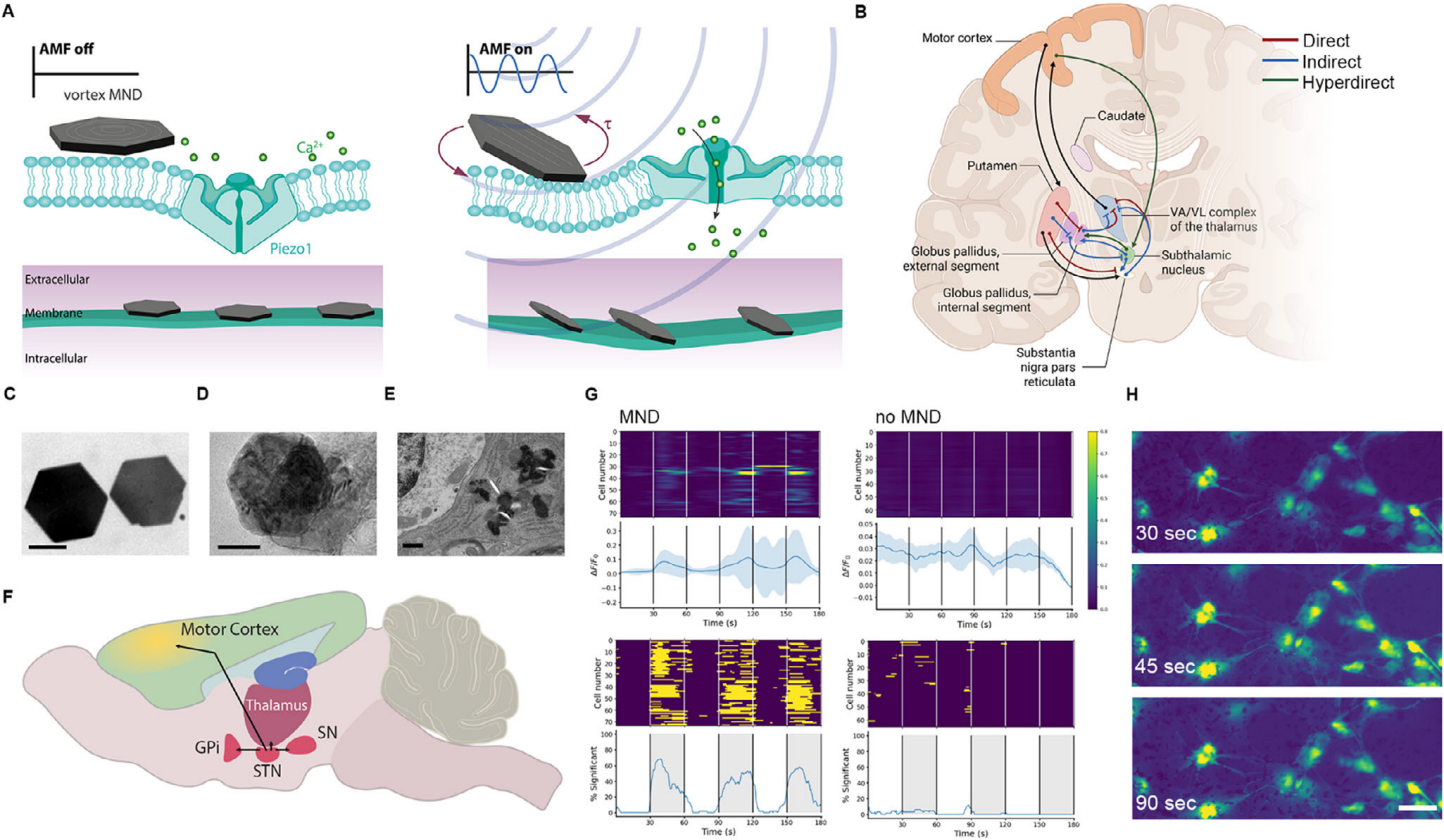
MNDs for magnetomechanical neuromodulation. (A) Illustration of MND‐mediated neuromodulation. (B) Schematic of the direct (red), indirect (blue), and hyperdirect (green) pathways under physiological conditions. Dopaminergic input from the substantia nigra pars compacta (SNc) activates the direct pathway and inhibits the indirect pathway. Cortical projections reach the subthalamic nucleus (STN) via the hyperdirect pathway, which then conveys signal to the globus pallidus internus (GPi). The GPi and substantia nigra pars reticulata (SNr) provide basal ganglia output, completing the cortico‐basal ganglia‐thalamo‐cortical pathway (adapted from [30]). (C) Transmission electron micrograph (TEM) of bare MNDs (100 nm). (D) TEM micrograph showing poly(maleic anhydride‐alt‐1‐octadecene)‐coated MNDs (100 nm). (E) TEM micrograph showing MND within the STN of a mouse (500 nm). (F) Illustration of a mouse brain highlighting the STN (pink) and analyzed regions: primary motor cortex (yellow), substantia nigra (pink), and ventral anterior/lateral thalamic nuclei (pink). (G) Heatmaps (top) showing fluorescence changes expressed as ΔF/F_0_ and significance plots (bottom) indicating the presence or absence of statistically significant response relative to baseline recorded from Fluo‐4 AM transients observed in human embryonic stem cell (hESC)‐derived neurons during magnetic field stimulus (grey) with (left) and without (right) MNDs. Each condition was repeated across six different cultures, with ∼10 cells randomly selected for analysis. (H) False‐color stills from a representative recording of hESC‐derived neurons (50 µm).

Here, we introduce magnetomechanical DBS (mDBS; Figure 1A), a wireless, minimally invasive neuromodulation strategy that employs biocompatible magnetite nanodiscs (MNDs; Figure 1C,D) [16] to activate mechanosensitive ion channels in the brain, such as Piezo‐type mechanosensitive ion channel component 1 (Piezo1) and transient receptor potential vanilloid 4 (TRPV4) [17, 18]. Unlike conventional DBS, mDBS delivers spatially confined stimulation through piconewton‐scale torques generated under low‐frequency AMF [16]. This enables precise, therapeutic modulation of deep brain circuits, thereby overcoming limitations of wired neurostimulation approaches. By eliminating implanted hardware, mDBS could extend access to DBS for patients who decline or are ineligible for surgery, using injectable, magnetic resonance imaging (MRI)‐compatible nanoparticles detectable with standard clinical imaging.

A single prior study reported magnetomechanical stimulation using MNDs [19], based on our earlier work on MND design and magnetic parameter optimization [16]. However, that approach relied on high frequency and high amplitude magnetic fields and targeted transient receptor potential cation channels, which likely limited efficacy and translational relevance [16]. Other magnetomechanical strategies, such as mTorquer, require extremely large nanoparticle concentrations (50 mg/mL) and genetic overexpression of Piezo1 to achieve functional efficacy, further constraining their clinical translation [20].

In contrast, the mDBS approach introduced here operates at clinically compatible field strengths to activate endogenous mechanosensitive ion channels such as Piezo1 and TRPV4, enabling targeted neuromodulation without genetic modification or high nanoparticle burden. Piezo1 and TRPV4 are expressed in neurons and glial cells in multiple regions of the mammalian central nervous system (CNS) [21, 22] and play a key role in transducing mechanical strain into electrical or biochemical responses [23, 24]. Although their role in glia and vascular mechanotransduction in the CNS is well established [22, 25, 26], their contribution to neural circuit dynamics remains largely unexplored.

Here, we harness these endogenously expressed mechanosensitive ion channels to enable precise and wireless neuromodulation using MNDs actuated by clinically relevant low‐frequency AMF parameters. We show that microgram‐level nanoparticle doses are sufficient to evoke robust neuromodulatory responses in vitro, ex vivo, and in freely moving mice. This approach reveals a functional role for endogenous mechanosensitivity in modulating neural activity and provides a genetically unmodified, clinically scalable paradigm for minimally invasive DBS.

Crucially, we demonstrated functional recovery in a severe Parkinsonian mouse model, representing the first therapeutic application of fully endogenous magnetomechanical neuromodulation. Our mDBS approach may offer potential for future human applications, with the possibility of improving patient adherence and clinical outcomes in DBS treatment [12].

## 2 Results

### 2.1 MNDs Mediate Calcium Influx In Vitro via Endogenous Mechanosensitive Ion Channels

We examined whether MNDs can induce intracellular calcium responses via magnetomechanical stimulation, thereby activating mechanosensitive ion channels in vitro. Human embryonic stem cell (hESC)‐derived neurons (H9 lineage, differentiated for >10 weeks) were incubated with the intracellular calcium indicator Fluo‐4 AM and exposed to AMF stimulation (5 Hz, 28 mT) [16]. Cells were treated with or without 30 µg/mL MNDs and subjected to three 30s AMF On/AMF Off cycles using a custom‐built solenoid coil (Figure 1A,C).

In cultures pre‐incubated with MNDs, AMF pulses consistently showed robust increases in Fluo‐4 fluorescence (ΔF/F_0_) (Figure 1G,H). No response was observed in MND‐free controls (Figure 1G), MND‐treated cultures that were not exposed to AMF (Figure S2D,E), or in the presence of the mechanosensitive cation channel inhibitor GsMTx4 (Figure S2F). Consistent with activation of mechanosensitive pathways, addition of the Piezo1 agonist Yoda1 induced an increase in Fluo‐4 fluorescence (Figure S2G), indicating that intracellular Ca^2+^ influx is mediated by mechanosensitive channels [27, 28, 29]. The safety of magnetomechanical actuation under these conditions was confirmed by the absence of propidium iodide uptake 30 min after AMF stimulation (Figure S2H). Immunohistochemistry confirmed the expression of Piezo1 and TRPV4 in differentiated hESC‐derived neurons (Figure S2I).

To assess the biocompatibility of the MNDs, we monitored HEK293 cell proliferation using phase‐object confluence measurements. Over five days, untreated HEK293 cells showed a steady increase in confluence, reaching approximately an eight‐fold increase (Figure S2A,B). Exposure to MND at concentrations of 15 and 30 µg/mL did not inhibit cell growth, indicating the absence of cytotoxic effects. These findings confirm that MNDs are well tolerated and do not impair HEK293 cell viability or proliferation at low concentrations. The concentration used for all further in vitro experiments was 30 µg/mL.

### 2.2 MND‐Mediated Magnetic Stimulation Evokes Neuronal Activation Ex Vivo

**FIGURE 2:**
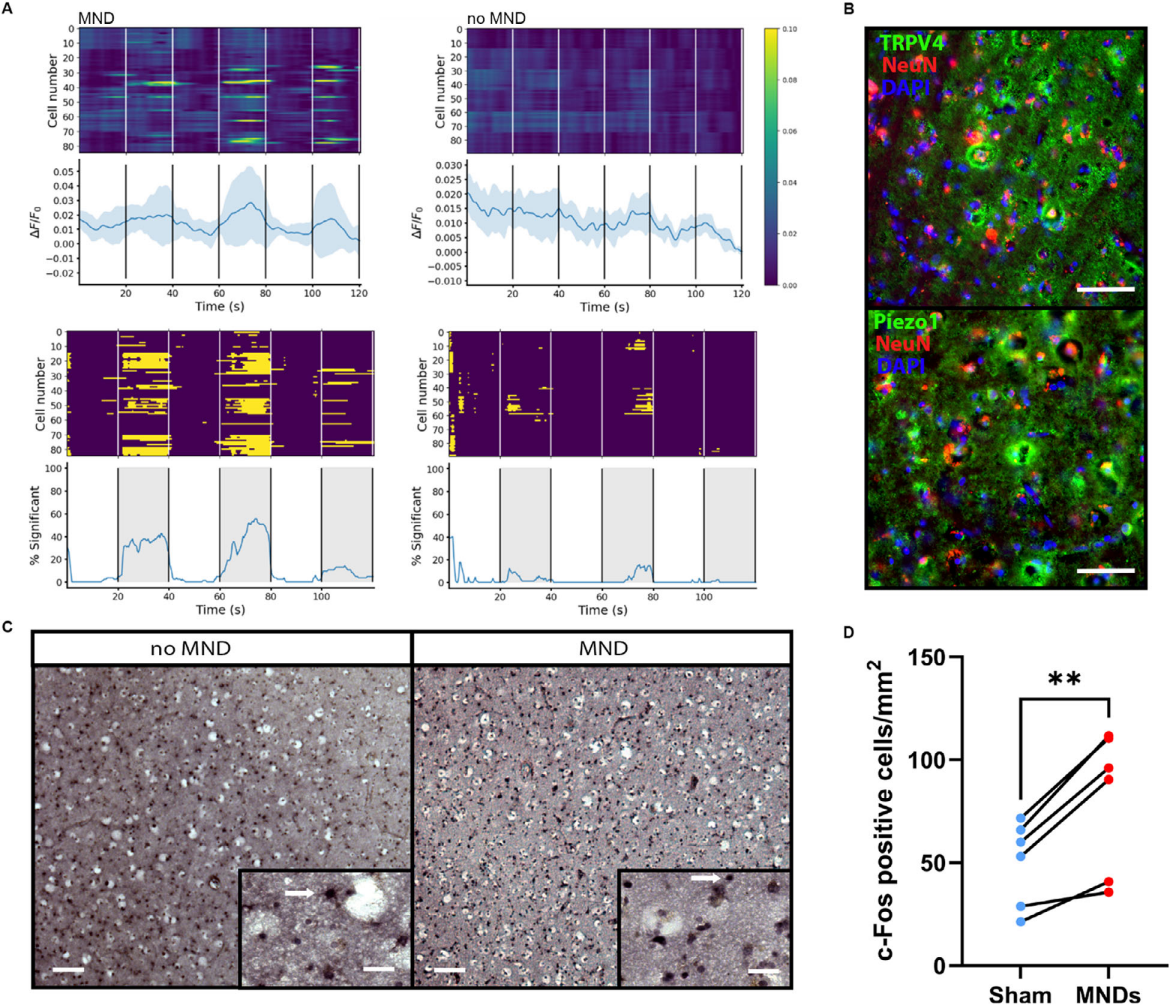
Ex vivo magnetomechanical control. (A) Heatmaps (top) showing fluorescence changes expressed as ΔF/F_0_ and significance plots (bottom) indicating the presence or absence of statistically significant response relative to baseline recorded from Fluo‐4 AM transients observed in hBSC during magnetic field stimulus with (left) and without (right) MNDs. Each condition was repeated across six different cultures, with ∼15 cells randomly selected per culture. (B) Representative high‐power (50 µm) photomicrographs of hBSC stained for NeuN with TRPV4 or Piezo1. (C) Representative low‐ (100 µm) and high‐power (25 µm inset) photomicrographs of hBSC stained for c‐Fos in slices receiving mDBS (*n* = 6) or control stimulation (*n* = 6). The white arrows indicate examples of c‐Fos‐positive cells. (D) Quantification comparing AMF‐only slices (*n* = 6) and mDBS‐treated slices (*n* = 6). mDBS significantly increased c‐Fos expression ^**^ *p* < 0.01, mean ± S.E.M., one‐tailed paired *t*‐test.

To address magnetomechanical activation in brain tissue, we incubated human organotypic brain slices (hBSCs; Table 1) 3 days post‐resection in artificial cerebrospinal fluid (aCSF) with ∼4 µg MNDs or without MNDs. Magnetomechanical stimulation was performed using the same custom‐built solenoid coil system (Figure S1A,C) with three 20s AMF On/ Off cycles. Calcium imaging revealed that only neurons treated with MNDs exhibited a reproducible increase in calcium signals during the field‐On epochs (Figure 2A; Figure S4), confirming effective mechanical stimulation in a physiologically relevant ex vivo environment. Immunohistochemistry confirmed the expression of Piezo1 and TRPV4 in human neurons (Figure 2B).

**TABLE 1:** Patient demographics and experimental applications.

Gender	Age	Experiment(s)
M	39	IHC; C‐Fos
F	31	IHC; C‐Fos
F	46	IHC; C‐Fos
F	32	IHC; C‐Fos
M	32	IHC; C‐Fos
F	56	IHC; C‐Fos
M	25	Ca^2+^‐Imaging
F	63	IHC; TRPV4 and Piezo1

To determine whether MND‐mediated stimulation activates functional neural circuits, tissues were fixed 90 min after AMF treatment and subsequently immunolabeled for c‐Fos, a marker of recent neuronal activity [31]. Slices incubated with MNDs and exposed to AMF showed a significant increase in c‐Fos expression compared to AMF‐only controls (t [5] = 5.4180, *p* = 0.0018, Figure 2C,D). These findings demonstrate that magnetomechanical actuation can effectively induce activity‐dependent gene expression in intact human brain tissues.

### 2.3 In Vivo MND‐Based Magnetomechanical Stimulation Modulates Motor Behavior In Mice

Next, we tested the ability of MNDs to modulate brain activity in vivo. Adult C57BL/6J mice received unilateral stereotaxic injections of 1.5 µg MNDs (1.5 µL per animal) into the subthalamic nucleus (STN) (Figure 1E), a deep brain structure clinically targeted for DBS in patients with PD. Unilateral targeting was chosen, given that classical unilateral STN DBS induces changes in motor behavior in rodents [32]. Motor behavior in response to magnetomechanical STN stimulation of mechanosensitive ion channels (Figure S3D,E) was evaluated 1–2 weeks post‐injection.

To compare AMF On and Off conditions, mice were placed inside the AMF coil for 3 min at 5 Hz and 28 mT. AMF stimulation led to significant improvement in motor performance on the rotarod task, which evaluates balance, coordination, physical condition, and motor planning. Mice exposed to AMF remained on the rotating rod for significantly longer than during the AMF Off sessions (t [9] = 2.708, *p* = 0.0120, Figure 4B). In addition, mice were evaluated using the open field test (OFT) for 5 min. The OFT assesses general locomotor behavior in a novel open arena. Following AMF On stimulation, mice exhibited significantly reduced movement compared to the AMF Off condition (t [10] = 1.860, *p* = 0.0463; Figure S5A–C).

To examine lateralized motor output, rotational behavior was evaluated in a circular plexiglass arena (Ø 9 mm, height 30 cm) fitted within a custom‐built solenoid coil (Figure S1D). Mice showed no significant change in contralateral rotations (t [20] = 1.2209, n.s., Figure S5D,E) or ipsilateral rotations (t [20] = 0.2868, n.s., Figure S5D,E) during the AMF On versus AMF Off conditions. While a trend toward reduced ipsilateral rotations was observed during the AMF On condition, the overall rotational symmetry remained unchanged.

**FIGURE 3:**
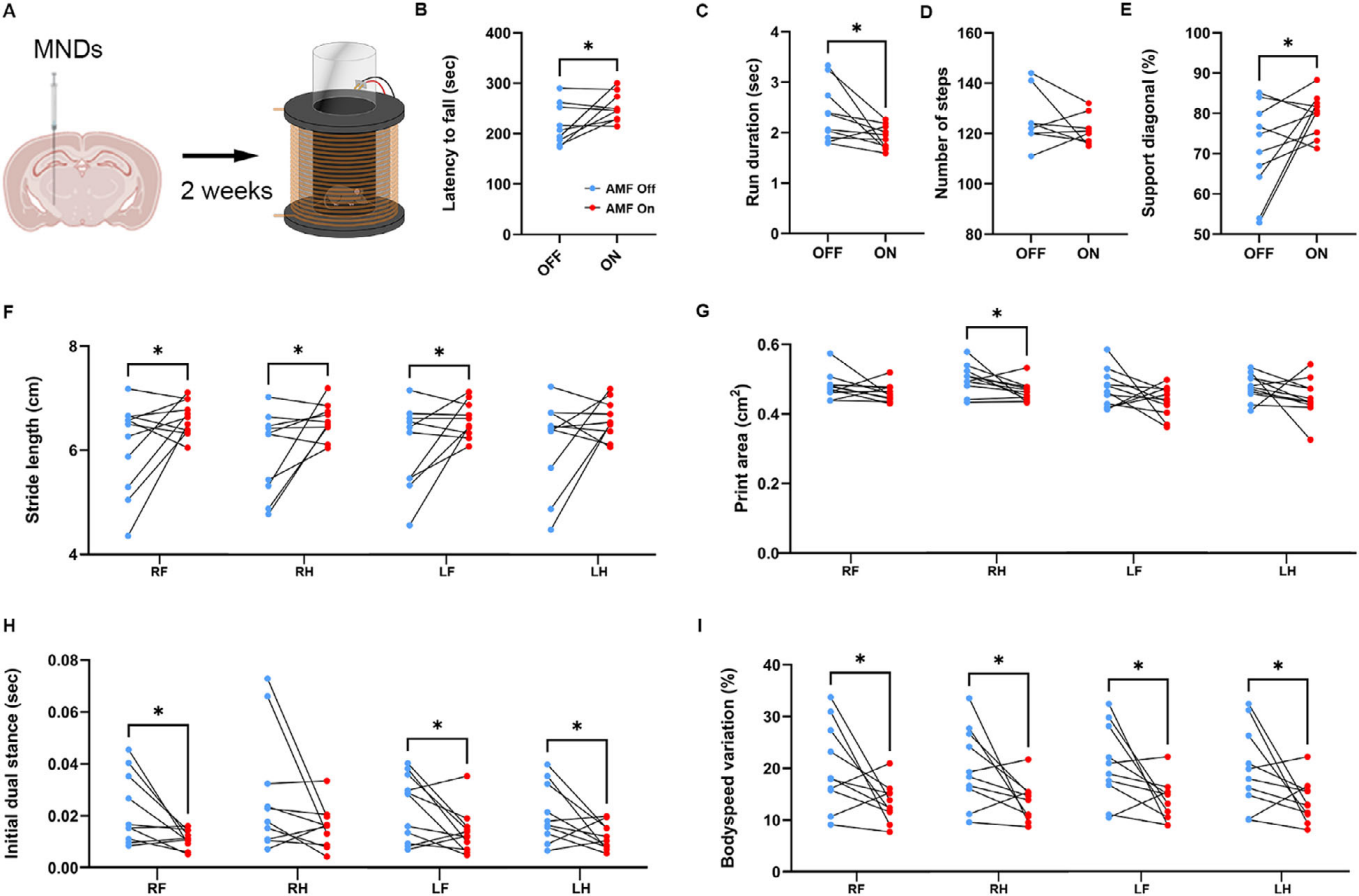
Magnetomechanical STN DBS improves motor behavior in naïve male mice. (A) In vivo experimental scheme. (B) Rotarod latency to fall across four trials during AMF Off and AMF On conditions. STN‐targeted MND stimulation delivered 3 min prior to testing improved motor performance (paired *t*‐test, t [9] = 2.708, ^*^ *p* = 0.0120). (C‐I) CatWalk XT gait metrics across five runs in AMF Off versus AMF On conditions (*n* = 11). AMF stimulation reduced run duration (^*^ *p* = 0.0173) (C), did not alter step number (n.s.) (D), increased diagonal limb support (^*^ *p* = 0.0275) (E), increased stride length (*p* ≤ 0.0346) (F), decreased paw print area (*p* = 0.0176) (G), decreased initial dual stance (*p* ≤ 0.0276) (H), and reduced body speed variation (*p* ≤ 0.0290) (I) of STN mDBS (*n* = 11) during AMF On and Off conditions (paired *t*‐test). ^*^ *p* < 0.05, mean ± S.E.M., RF: right front paw, RH: right hind paw, LF: left front paw, LH: left hind paw.

AMF stimulation also produced significant improvements in gait‐ and balance‐related general coordination, as assessed using the CatWalk XT test [33]. Mice exhibited decreased run duration (*p* = 0.0173, Figure 3C), no change in the number of steps (Figure 3D), increased diagonal support (*p* = 0.0275, Figure 3E), and increased stride length (*p* ≤ 0.0346, Figure 3F). Additional changes included a decreased print area (*p* = 0.0176, Figure 3G), decreased initial dual stance (*p* ≤ 0.0276, Figure 3H), and lower body speed variation (*p* ≤ 0.0290, Figure 3I).

Supplementary analyses revealed a decreased maximum contact area (*p* ≤ 0.0255) (Figure S8A), decreased terminal dual stance (*p* ≤ 0.0425) (Figure S8B), decreased print length (*p* = 0.0026) (Figure S8C), unchanged print width (Figure S7D), unchanged speed duty cycle (Figure S7E), decreased run max variation (*p* = 0.0075) (Figure S8F), and decreased support three (*p* = 0.0134) (Figure S8G), indicating enhanced dynamic coordination and postural control following AMF stimulation.

Consistent with in vitro effects (Figure S2A), in vivo exposure to MNDs showed no increase in astrocytes and microglia. In the MND‐injected hemisphere (*n* = 7), no significant increase in microglia was found compared to the non‐injected hemisphere (t [6] = 0.4114, n.s.). Similarly, no significant increase in astrocytes was found in the MND‐injected hemisphere (*n* = 8) compared to the non‐injected hemisphere (t [7] = 0.3581, n.s.) (Figure S3).

### 2.4 Therapeutic Efficacy of Magnetomechanical DBS in a Severe Hemiparkinsonian Mouse Model

**FIGURE 4:**
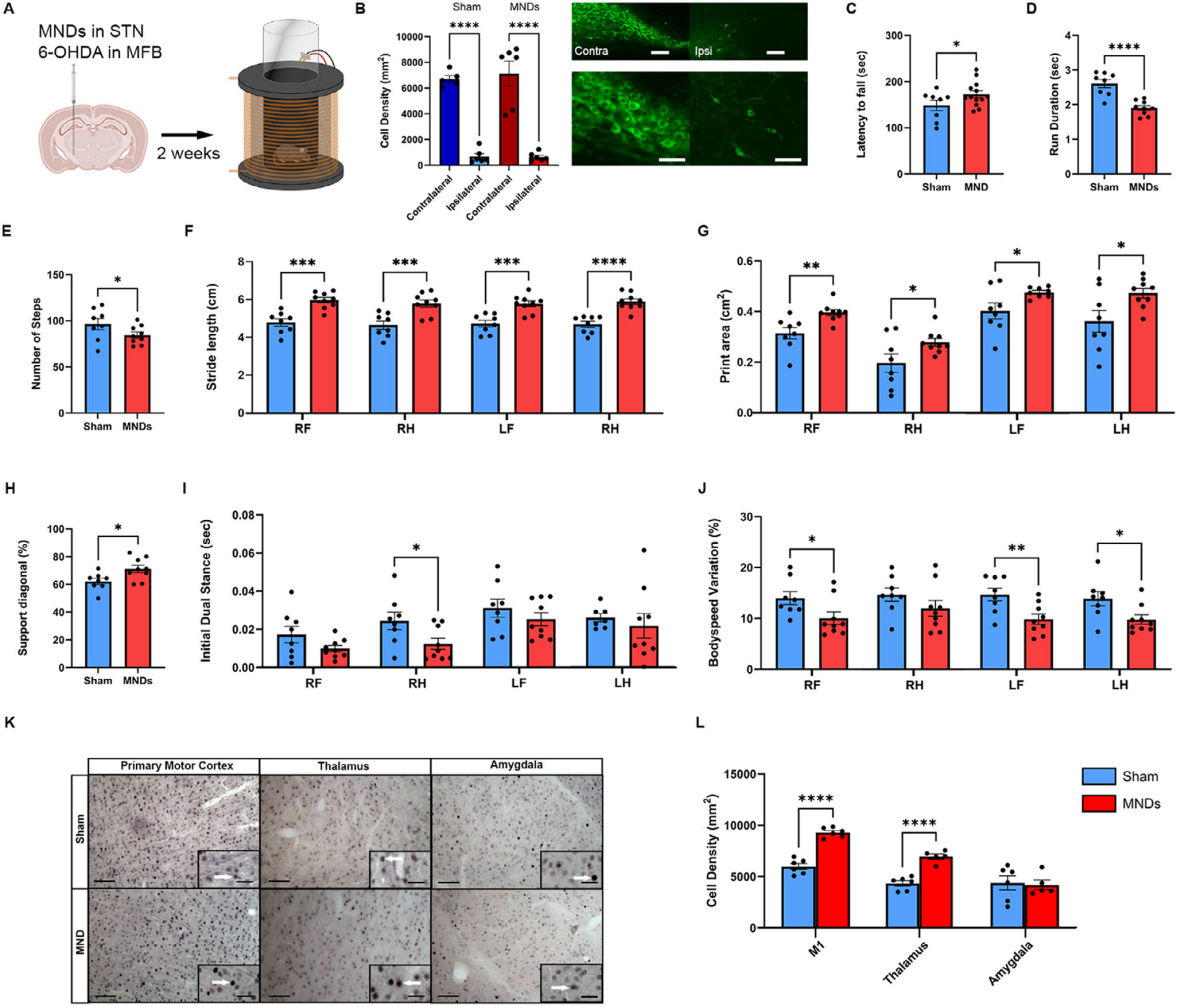
Magnetomechanical STN DBS alleviates severe Parkinsonian symptoms. (A) In vivo experimental scheme. (B) Representative low‐ (1000 µm) and high‐power (50 µm) photomicrographs of Tyrosine Hydroxylase (TH)‐positive cells in the substantia nigra pars compacta (SNc) contralateral and ipsilateral to the 6‐OHDA injection. The lesion caused ∼90% loss of TH in the ipsilateral SNc versus the contralateral side (mean ± S.E.M., one‐way ANOVA), ^****^ *p* < 0.0001. (C) Rotarod performance across five trials on two testing days. STN‐targeted MND stimulation improved motor performance versus sham (t [19] = 1.875, *p* = 0.0381). (D–J) CatWalk XT results across four runs. AMF stimulation reduced run duration (*p* < 0.0001) (D) and step count (*p* = 0.0472) (E), increased stride length (*p* ≤ 0.0006) (F), decreased paw print area (*p* ≤ 0.0224) (G), increased diagonal limb support (*p* = 0.0118) (H), decreased initial dual stance (*p* = 0.0190) (I), and decreased body speed variation (*p* = 0.0352) (J) between sham (*n* = 9) and STN mDBS mice (*n* = 8), independent one‐tailed *t*‐test. (K) Representative low‐ (500 µm) and high‐power (50 µm) c‐Fos photomicrographs in M1, ventral anterior/ lateral thalamus, and amygdala in sham (*n* = 6) and STN mDBS (*n* = 6). The white arrows indicate examples of cells expressing c‐Fos. (L) STN mDBS increased c‐Fos in M1 (*p* < 0.0001) and thalamic motor areas (*p* < 0.0001), but not in the amygdala. ^*^ *p* < 0.05, ^**^ *p* < 0.01, ^***^ *p* < 0.001, ^****^ *p* < 0.0001, mean ± S.E.M., RF: right front paw, RH: right hind paw, LF: left front paw, LH: left hind paw.

To assess the clinical relevance of mDBS, we applied this approach to a well‐established model of PD, using mice with a unilateral 6‐hydroxydopamine (6‐OHDA) lesion targeting the nigrostriatal pathway and projecting to the STN. Given that STN stimulation is a standard DBS target for levodopa‐resistant motor complications, MNDs were unilaterally injected into the STN of hemiparkinsonian mice. This hemiparkinsonian model is characterized by selective dopaminergic neuron loss on one side of the brain, mimicking the motor deficits observed in PD [34]. We found that a unilateral 6‐OHDA injection resulted in a 90% reduction in Tyrosine Hydroxylase (TH) expression in the ipsilateral substantia nigra pars compacta (SNc) when compared to the contralateral hemisphere (*p* < 0.0001) (Figure 4B). To compare the behavioral effects of mDBS, sham, and MND‐treated mice were evaluated for their performance on the rotarod. Performance was significantly improved in the MND group compared to the sham control group (t [19] = 1.875, *p* = 0.0381) (Figure 4C), indicating improved balance, coordination, physical condition, and motor planning under magnetomechanical stimulation.

Rotational behavior was quantified as previously described by assessing ipsilateral or contralateral rotations around the body axis. While a trend toward reduced ipsilateral turning was observed in MND‐treated mice, statistical analyses revealed no significant differences in ipsilateral (t [20] = 0.2868, n.s.) (Figure S6A) or contralateral rotations (t [20] = 0.2868, n.s.) (Figure S6B) between sham and STN mDBS. Nevertheless, the overall distribution of rotational behavior appeared to be more balanced under stimulation, suggesting subtle modulatory effects.

Sham and MND‐treated mice were additionally evaluated in the OFT for 5 min as previously described. No significant difference in the total distance travelled was observed between the MND and sham groups (t [15] = 0.1679, n.s.) (Figure S7A), although a positive trend toward increased locomotion was observed in the MND group. Similarly, the average velocity did not differ between groups (t [14] = 0.9838, n.s.) (Figure S7B). The time spent in the center (t [15] = 1.142, *p* = 0.1356) (Figure S7C), corners (t [15] = 0.9593, n.s.) (Figure S7D), and along the walls (t [15] = 0.08598, n.s.) (Figure S7E) of the OFT arena were also comparable between the groups, indicating no anxiety‐related behavior (Figure S7).

In the CatWalk XT test, AMF stimulation induced significant changes in multiple gait and balance‐related parameters, reflecting improved coordination and motor control. MND‐treated mice exhibited a decreased run duration (*p* < 0.0001) (Figure 4D), reduced number of steps (*p* = 0.0472) (Figure 4E), increased stride length (*p* ≤ 0.0006) (Figure 4F), increased print area (*p* ≤ 0.0224) (Figure 4G), and diagonal support (*p* = 0.0118) (Figure 4H). The contact dynamics also changed with an elevated maximum contact area (*p* ≤ 0.0228) (Figure S9A), increased terminal dual stance (*p* ≤ 0.0246) (Figure S9B), and a higher initial dual stance (*p* = 0.0190) (Figure 4I). Additional improvements included increased print dimensions, length (*p* ≤ 0.0110) (Figure S9C), and width (*p* ≤ 0.0358) (Figure S9D). Measures of gait variability were reduced, including decreased body speed variation (*p* = 0.0352) (Figure 4J), speed duty cycle (*p* = 0.0352) (Figure S9E), and maximum run variation (*p* = 0.0485) (Figure S9F), consistent with enhanced dynamic stability and postural control.

Consistent with behavioral effects, magnetomechanical stimulation activated neural circuits across motor regions. In AMF‐stimulated mice (*n* = 6), a significantly higher proportion of c‐Fos positive cells was detected in the motor cortex (t [10] = 9.405, *p* < 0.0001) and ventrolateral and ventral anterior thalamic complex (t [9] = 6.958, *p* < 0.0001) compared to the sham control group (*n* = 6). No difference was observed in the amygdala (t [9] = 0.2276, n.s.) in AMF‐stimulated mice (*n* = 5) when compared to the sham control group (*n* = 6) (Figure 4K,L), confirming the effective activation and recruitment of areas of the cortico‐basal ganglia‐thalamo‐cortical circuitry (Figure 1B,F). The degree of motor recovery observed parallels improvements achieved with STN‐DBS in advanced PD, supporting the translational potential of mDBS as a minimally invasive, electrode‐free neuromodulation strategy.

### 2.5 Magnetomechanical STN Stimulation Drives Network‐Level Neural Responses In Vivo

**FIGURE 5:**
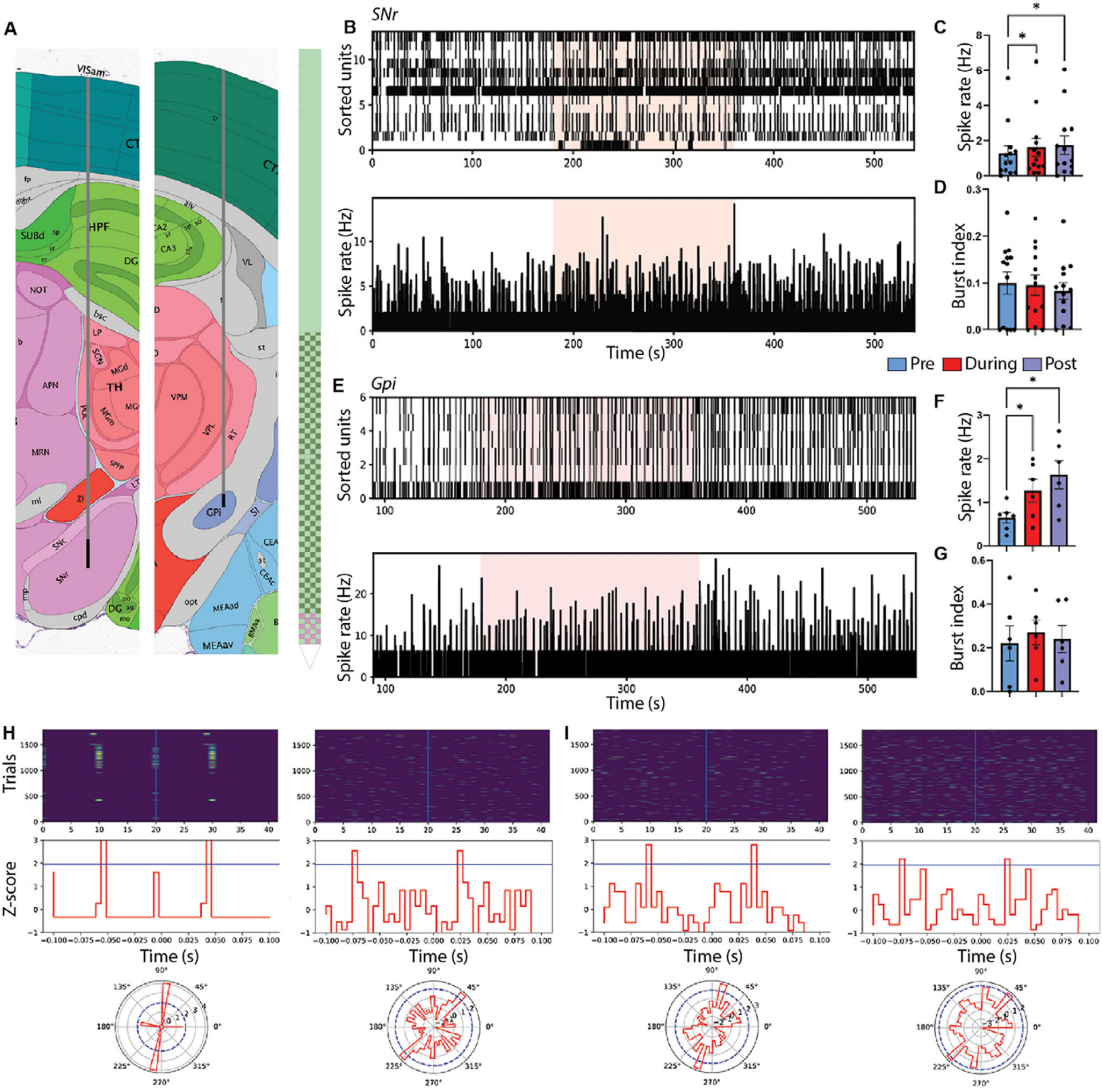
Magnetomechanical STN DBS neural responses. (A) Neuropixels probe trajectories targeting SNr (pink) and GPi (blue) based on the Allen mouse brain atlas, with recording sites shown. (B) Raster plot (top) of 13 units recorded before, during, and after AMF stimulation with burst index (bottom) shown as the summed spike rate of all units, binned at 50 ms intervals in the SNr. (C) Average spike rates of the 13 SNr units significantly increased (*p* < 0.05) during and after AMF stimulation compared to the pre‐stimulation baseline. (D) Burst index remained unchanged during and after AMF stimulation relative to the pre‐stimulation period. (E) Raster plot (top) of 6 units recorded before, during, and after AMF stimulation with burst index (bottom) shown as the summed spike rate of all units, binned at 50 ms intervals in the globus pallidus internus. (F) Average spike rates of the 6 GPi units significantly increased (*p* < 0.05) during and after AMF stimulation compared to the pre‐stimulation baseline. (G) The average burst index remained stable during and after AMF stimulation relative to the pre‐stimulation period. (H) Two representative substantia nigra units responding to AMF stimulation. Top: trial‐aligned raster plots showing spike activity 0.1 s before and after AMF onset. Bottom: polar plots showing preferred phase of AMF modulation; blue line denotes Z‐score threshold of 1.96. (I) Two representative globus pallidus internus units responding to AMF stimulation. ^*^ *p* < 0.05, mean ± S.E.M. Repeated‐measures ANOVA with Bonferroni post hoc correction.

To investigate the effects of low‐frequency magnetomechanical activation of the STN on basal ganglia output and characterise network‐level neural responses in vivo, we performed high‐density extracellular recordings targeting the SNr and GPi in a hemiparkinsonian mouse model (Figure 5A). Spike sorting and assignment of single units were based on stereotactic coordinates and confirmed histologically. During electrophysiological recordings, repeated measures ANOVA revealed a significant effect of AMF stimulation on the firing rate of SNr neurons across baseline, during, and after stimulation (F [2, 12] = 6.802, *p* = 0.0063) (Figure 5B,C). Further post‐hoc comparisons revealed a significant increase in firing rate between baseline and stimulation (*p* = 0.0430) and between baseline and post‐stimulation (*p* = 0.0280). At the same time, no difference was found between stimulation and post‐stimulation. Similar results were observed in the GPi, with repeated measures ANOVA indicating a significant effect across time points (F [2, 5] = 14.25, *p* = 0.0071) (Figure 5E,F). In particular, a significant increase in firing rate was observed between baseline and stimulation (*p* = 0.0302) and between baseline and post‐stimulation (*p* = 0.0320), while no difference was found between stimulation and post‐stimulation. These findings suggest that low‐frequency magnetomechanical stimulation can modulate basal ganglia output in a manner comparable to conventional high‐frequency STN‐DBS [35], potentially preserving or restoring more physiological neural activity patterns within the network.

Interestingly, SNr and GPi neurons showed no changes in bursting activity during AMF stimulation (Figure 5D,G). Bursting activity in the basal ganglia nuclei is a well‐established electrophysiological hallmark of PD, due to dopamine depletion, and is typically associated with impaired motor function [36, 37, 38]. Conventional high‐frequency STN DBS suppresses bursting activity [38]. Therefore, our findings suggest that AMF stimulation modulates neuronal firing rates without affecting pathological bursting patterns, indicating a distinct mechanism of action compared to conventional high‐frequency DBS.

We next investigated whether neuronal activity in basal ganglia output structures was entrained by AMF stimulation. Representative single‐unit recordings from the SNr revealed time‐locked responses, with spikes aligned to stimulation onset showing changes in firing within a 100‐ms window surrounding the event (Figure 5H, top). Phase analysis further revealed coherent entrainment and significant spike‐AMF phase locking in both the SNr and GPi (Figure 5H,I), with preferred phases exceeding the statistical threshold (Z = 1.96).

## 3 Discussion

There is an increasing interest in advanced neuromodulation technologies for PD, particularly in cases where pharmacological treatments are insufficient. STN‐DBS has proven to be more effective for advanced PD, demonstrating increased efficacy over dopaminergic medication alone in managing motor symptoms and improving the quality of life in patients [39]. The STN plays a central role in the cortico‐basal ganglia‐thalamo‐cortical circuitry, exerting excitatory glutamatergic projections to downstream nuclei, including the globus pallidus and substantia nigra [40, 41]. However, analyses of DBS surgical outcomes have found that 15%–34% of DBS surgeries later require follow‐up surgical interventions to either remove or revise the placement of DBS electrodes. Of these, 47% were due to hardware malfunctions or infections.

Additionally, minor surgery is required regularly for battery replacement in the pacemaker component of DBS devices [42]. These risks associated with conventional DBS contribute to its underutilization, as a significant number of patients decline the procedure due to surgery‐related concerns, resulting in a mere 10% of eligible patients ultimately receiving DBS [43]. Given this, there is an increasing demand for neuromodulatory approaches that offer greater specificity, improved safety, and long‐term stability, while minimizing invasiveness. Recent innovations in miniaturized and wireless systems have sought to address these limitations by enabling remote neural control without the need for chronically implanted hardware or frequent surgical interventions such as battery replacement [8, 12]. For clinicians, the ability to deliver DBS‐like therapeutic benefits without implanted hardware could eliminate device‐related infections, lower revision surgery rates, and enable earlier intervention in disease progression.

Within this context, magnetothermal DBS has emerged as a promising strategy for remote neuromodulation through externally applied magnetic fields [12, 44, 45]. However, to date, no clinical studies targeting neurological disorders have been reported. This is largely due to limitations such as reliance on transgenes, low temporal resolution, and high nanoparticle concentrations [12, 13]. Recently, approaches capable of delivering mechanical or electrical stimuli without genetic modification have therefore gained attention due to their translational potential [8, 9, 14, 16].

In this study, we introduce mDBS, a wireless approach using injectable, anisotropic MNDs with vortex magnetization that transduce externally applied AMF energy into mechanical forces to activate neurons in the STN. After characterizing the magnetization behavior and confirming previously demonstrated biocompatibility of MNDs stabilized with poly(maleic anhydride‐alt‐1‐octadecene) (PMAO) and their non‐toxic effects, we validated the mechanistic underpinnings in in vitro models.

Through Ca^2+^ imaging in hBSC and hESC‐derived neurons, we observed that MND stimulation induced transient calcium influx through mechanosensitive ion channels, predominantly mediated by Piezo1. Pharmacological inhibition of Piezo1 and TRPV4 using GsMTx4 significantly reduced AMF‐induced activity, whereas the Piezo1 agonist Yoda1 potentiated responses in vitro. Together, these results confirm that MND‐driven AMF exposure causes torques sufficient for membrane deformation and Piezo1 opening, initiating a calcium influx cascade, similar to our reported MND application to activate dorsal root ganglions dominated by Piezo2, with ∼18 pN exerted from a single MND under 5 Hz and 28 mT AMFs [16].

Immunohistochemical analysis further revealed the presence of Piezo1 and TRPV4 in ex vivo brain tissue, suggesting the possibility of multi‐channel mechanotransduction. Although our functional experiments focused on Piezo1, TRPV4 is known to respond to mechanical and osmotic stimuli and is involved in neurosensory and glial modulation [24, 48]. Its expression raises the possibility of parallel or synergistic activation of pathways during mDBS. However, TRPV4 activation often requires more sustained or indirect stimuli compared to Piezo1's direct bilayer tension gating [23]. Future studies using TRPV4 antagonists or knockout models may reveal whether this channel contributes to neuromodulatory outcomes, particularly under chronic stimulation. Importantly, the presence of multiple mechanosensitive channels highlights the potential of mDBS to engage a broader range of endogenous mechanosensitive pathways, enhancing its robustness and translational scope.

In naïve mice, unilateral stereotactic injection of MNDs into the STN, followed by AMF stimulation, significantly enhanced performance in the rotarod and CatWalk XT assays. These findings suggest that mDBS can modulate motor circuits, even under baseline physiological conditions, thereby increasing motor coordination and balance. Notably, significant improvements in gait and balance assays, including walking speed, stride length, and variability in body speed, support the notion that magnetomechanical activation selectively enhances locomotor function, likely via motor‐related neuronal pathways, including the STN and its downstream effects on the globus pallidus externa (GPe) and SNr via the indirect and hyperdirect pathways of the basal ganglia [49], as well as the motor thalamus [50], motor cortex [49], and brain stem centers, such as the pedunculopontine nucleus [51].

To test its therapeutic potential, mDBS was applied in a severe 6‐OHDA hemiparkinsonian mouse model, which mimics advanced PD pathology with over 90% loss of TH‐positive neurons in the SNc. While general locomotor activity in the OFT did not improve, likely because of the limited sensitivity of the test in detecting subtle motor improvements in severe dopaminergic depletion, mice treated with mDBS displayed significant benefits in rotarod latency and CatWalk XT parameters including increased walking speed, extended stride length, and a reduced duty cycle, suggesting a more efficient gait with less time spent in the stance phase. These results mirrored those observed in naïve animals. The behavioral improvements also align with key clinical endpoints used in DBS trials, such as gait speed, balance, and coordination, which underscores the direct clinical relevance of the magnetomechanical response.

Static CatWalk XT parameters also showed improvements compared to the sham controls. These behavioral outcomes are consistent with those of conventional STN DBS studies, including enhanced forelimb use asymmetry [52], enhanced treadmill performance [53], stepping and rotarod scores [54], and increased locomotor activity [55]. Notably, static and dynamic Catwalk XT parameters have differential sensitivity to stimulation‐induced changes in locomotor speed, suggesting that the dynamic improvement observed here reflects the selective engagement of fast locomotor circuits by mDBS.

To assess downstream neuronal activation, c‐Fos immunostaining was used as a marker of neural activity [31]. In hBSC, AMF stimulation in the presence of MNDs significantly increased c‐Fos‐positive cell counts compared to AMF alone. In vivo, a similar increase in c‐Fos‐positive cells was observed in the motor cortex and motor thalamus, confirming that MND‐mediated mDBS can wirelessly drive cellular responses in mechanosensory deep brain structures. Although hBSCs offer a translational ex vivo platform, some limitations need to be considered, particularly the fact that this tissue is typically resected from pharmacoresistant epilepsy patients. While this does not entirely exclude the impact of disease‐related changes, several factors support the broader relevance of our results. First, slices were obtained from cortical regions distant from the epileptogenic area, thereby reducing the epileptogenic effects. Second, our findings were confirmed in vivo, which are free from epileptic pathology, strengthening the generalizability of the observed neuromodulatory effects. Importantly, this study integrates human ex vivo tissue and mouse in vivo models, allowing complementary levels of validation. Human brain slices were used to directly validate magnetomechanical activation of endogenous mechanosensitive pathways in clinically relevant tissue, while mouse models enabled circuit‐level and behavioral assessment. The targeted mechanotransduction mechanisms, including Piezo1‐mediated force sensing and membrane‐cytoskeleton coupling, are highly conserved across species, supporting mechanistic continuity between the human and mouse data [59, 60].

To examine the magnetomechanical activation of the STN, we performed high‐density extracellular recordings targeting the SNr and GPi in a hemiparkinsonian mouse model. AMF stimulation resulted in modulation of the basal ganglia output by increasing the firing rates in both structures. These increases were observed during and after stimulation, suggesting that mDBS modulates downstream basal ganglia output pathways. This effect is similar to the response to conventional high‐frequency STN‐DBS, which increases tonic firing in the SNr and GPi while disrupting pathological synchrony [35]. Moreover, the increased excitability is in line with previous research indicating that 6‐OHDA lesions disrupt the balance of synaptic inputs in favor of excitation, particularly in the GPi [56].

Despite the increase in firing rate, bursting activity in the SNr and GPi neurons remained unchanged during and after stimulation. Bursting is a hallmark of Parkinsonian basal ganglia activity and is closely related to dopamine depletion and motor dysfunction [36, 37, 38]. While high‐frequency DBS is known to suppress pathological bursting [38], MND‐mediated stimulation did not alter this activity under the current parameters. This suggests that although magnetomechanical neuromodulation can modulate basal ganglia output, its mechanism of action may differ from that of conventional DBS. In addition, the ability of mDBS to impose phase‐locked activity on basal ganglia output structures suggests temporally entrainment, which could be applied to disrupt pathological oscillations in Parkinsonian circuits [57]. Together, these findings highlight the potential of mDBS to influence basal ganglia circuitry that is similar, but mechanistically distinct, from conventional DBS approaches [35, 41, 58].

Neuropixels recordings from basal ganglia output nuclei provide a circuit‐level readout integrating excitatory and inhibitory inputs. The consistent increase in SNr and GPi firing rates and spike‐AMF phase locking argue against canceling mixed activation and support coordinated modulation of therapeutic networks. A detailed cell‐type‐specific dissection of excitatory and inhibitory neuronal populations is an important focus of future studies.

In summary, this study presents a wireless, minimally invasive platform consisting of micromolar concentrations of biocompatible MNDs (1.5 µg per mouse) and simple, scalable solenoid coils for deep brain neuromodulation. By leveraging the magnetomechanical actuation of endogenous mechanosensitive ion channels dominated by Piezo1, mDBS enables targeted activation of the STN with high spatiotemporal resolution and without the use of genetic tools or implanted hardware. Together, the behavioral and histological data support the conclusion that MND‐mediated mDBS can effectively modulate neuronal activity in deep brain regions and induce functional changes. We propose that these effects are driven by selective activation of the thalamocortical circuitry, offering a minimally invasive, wireless neuromodulation strategy with therapeutic relevance for neurodegenerative diseases, such as PD. From a translational perspective, due to the ease of scaling and manipulating electromagnetic setups, upcoming work focuses on adapting coil geometries and field parameters for human brain volumes and assessing MND clearance and long‐term biocompatibility in large animal models. Combined with MRI‐guided injection techniques, this platform could be rapidly adapted for minimally invasive clinical feasibility trials in movement disorders.

## 4 Materials and Methods

### 4.1 Magnetic Nanodiscs

The MNDs were synthesized through a two‐step process. First, hematite nanodiscs were produced via hydrothermal synthesis. These were then reduced under hydrogen reflux at 360°C while dispersed in triethanolamine, which served as a co‐reducing agent, and oleic acid, employed as a dispersing agent. Oleic acid absorbs onto the MNDs, conferring hydrophobicity and providing new binding sites for subsequent functionalization with PMAO, applied at a concentration of 10 mg of PMAO per mg of MNDs, to enhance hydrophilicity, water dispersibility, and biocompatibility. Exposure to an AMF at a therapeutically relevant frequency of 5 Hz and an amplitude of 9 Vpp, resulting in the generation of a magnetic field amplitude of 28 mT, is sufficient to trigger the reversible activation of mechanosensitive ion channels through the torque produced by the MNDs.

### 4.2 Cell and Tissue Culture

#### 4.2.1 HEK293

HEK293 cells (ATCC CRL‐1573) were cultured in EMEM (Gibco; 11095080) supplemented with 10% fetal bovine serum, maintained at 37°C in a humidified atmosphere with 5% CO_2_. The cells were passaged every 3–4 days. Cells were seeded at 1.5 × 10^4^ cells/well in 96‐well plates for toxicity analysis, and 1% penicillin‐streptomycin was added to the cell culture medium.

#### 4.2.2 H9 Differentiated Human Neurons

H9 human embryonic stem cells (hESCs) obtained from WiCell were successfully expanded and confirmed to have a normal karyotype. The expression of pluripotency markers was validated following the protocol described by Marchetto et al. [61]. H9 cells were cultured on Geltrex‐coated plastic plates (Thermo Fisher Scientific) and maintained in E8 Flex medium (Thermo Fisher Scientific). Neural progenitor cells were generated using the STEMdiff Neural Induction Medium (Stemcell Technologies), according to the manufacturer's instructions. Neural progenitor cells were maintained in STEMdiff Neural Progenitor Medium and subsequently matured into neurons using Brainphys medium, supplemented with growth factors.

In brief, hESCs were enzymatically dissociated using Gentle Dissociation Reagent (Stem Cell Technologies) and plated as single cells (3 × 10^6^ cells/mL) in AggreWell 800 microwell culture plates (Stem Cell Technologies) containing STEMdiff Neural Induction Medium, supplemented with SMAD inhibitors and y‐27632, to generate EBs. Developing EBs were maintained in STEMdiff Neural Induction Medium supplemented with SMAD inhibitors for five days. After this period, the EBs were transferred to plates coated with polyornithine and laminin. Rosette‐forming EBs were selectively isolated using an enzyme‐free neural rosette selection reagent (Stem Cell Technologies) and plated on polyornithine‐laminin‐coated 35 mm dishes to generate neural progenitor cells (NPCs). These NPCs were cultured as high‐density monolayers and seeded at low densities (4 × 10^4^ cells/cm^2^) for neuronal differentiation.

During neuronal differentiation, NPCs were cultured in neural differentiation medium supplemented with brain‐derived neurotrophic factor (20 ng/mL, Peprotech), glial cell‐derived neurotrophic factor (20 ng/mL, Peprotech), dibutyryl‐cyclic AMP (1 mm, Sigma), ascorbic acid (200 nm, Sigma), and laminin (1 µg/mL) in BrainPhys Neuronal Medium (Stem Cell Technologies), which included N2 Supplement‐A and SM1. The culture medium was refreshed twice a week throughout the ten‐week differentiation process. Quality control was maintained to ensure that all cultures were free from mycoplasma contamination.

#### 4.2.3 Human Organotypic Brain Slice Cultures

Slices for organotypic culture were prepared from the temporal cortex of eight patients (three male and five female, aged 25–63 years; Table 1) diagnosed with pharmacoresistant temporal lobe epilepsy. All patients provided written informed consent, and the protocols were approved by the Medical Ethics Review Committee (METC 2024‐0496). Tissue processing followed a modified protocol based on the method described by Bak et al. [62]. Tissue was transported from the operating room to the laboratory in slicing artificial cerebral spinal fluid (s‐aCSF) composed of 110 mm choline chloride, 26 mm NaHCO_3_, 1.25 mm Na_2_HPO_4_, 11.6 mm sodium ascorbate, 3.1 mm sodium pyruvate, 7 mm MgCl_2_, 0.5 mm CaCl_2_, 2.5 mm KCl, 10 mm glucose, and 1% penicillin/streptomycin/amphotericin B in demineralized water. The solution was carbonated with 95% oxygen and 5% CO_2_ for at least 20 min before tissue resection.

Under sterile conditions, the tissue was washed with carbonated s‐aCSF, followed by careful dissection to remove meninges, capillaries, and damaged sections from the tissue block. Approximately 300 µm thick, cortical tissue slices were cut using a vibratome (Leica VT1200, Leica Biosystems, Wetzlar, Germany), with each slice measuring approximately 5 × 5 mm. These slices were placed on 0.4 µm pore‐size 6‐well cell culture inserts (CellQART 9300414, SABEU, Germany), creating an air‐liquid interface using intermediate step HEPES medium (ISHM).

ISHM was prepared by combining 23.5 mL DMEM F‐12 and 23.5 mL Neurobasal medium, supplemented with 1 mL B27, 0.5 mL N_2_ supplement, 0.5 mL GlutaMAX, 0.5 mL penicillin/streptomycin, and 0.5 mL non‐essential amino acids, yielding a total of 50 mL. To enhance the buffering capacity, 20 mm HEPES was added, and the mixture was stirred for at least 20 min. The slices were maintained at 37°C and 5% CO_2_. After 1 h, the slices on the inserts were transferred to a pre‐equilibrated 6‐well plate in an incubator containing artificial cerebrospinal fluid (aCSF). The aCSF composition included 125 mm NaCl, 25 mm NaHCO_3_, 2.5 mm KCl, 1.25 mm NaH_2_PO_4_, 1 mm MgCl_2_·6H_2_O, 2 mm CaCl_2_, 25 mm glucose, and 1% penicillin/streptomycin/amphotericin B in demineralized water. The aCSF was replaced two to three times per week. Tissue was afterward collected in 4% paraformaldehyde (PFA) for 24 h at 4°C. The tissue was then transferred to a 20% sucrose solution for a minimum of 24 h. The tissue was then fresh‐frozen and stored at ‐80°C.

#### 4.2.4 Cell Toxicity Analysis

MNDs were suspended in cell culture medium at 15, 30, 60, and 120 µg/mL and then applied to the cells. Growth of HEK293 cells over 5 days post‐MND administration was evaluated using the IncuCyte S3 imager (Sartorius, USA) with the basic analysis module. Phase area confluence values were normalized to the 0 h time point for each condition and presented as a ratio to quantify relative changes over time.

#### 4.2.5 In Vitro and Ex Vivo Magnetomechanical Stimulation

The magnetic stimulation setup was custom‐designed to fit into a Leica DIVI LFS microscope (Leica, Wetzlar, Germany) and accommodate a 35 mm dish. The system utilizes a magnetic coil to generate an alternating current (AC) magnetic field focused along the center of the cell culture well. A 1.32 mm‐thick copper wire was wound around a plastic coil frame with 990 turns. This magnetic solenoid coil, used for the in vitro and ex vivo parts in this study, was custom‐built in our lab by IDEE Research Engineering (based on the design of Gregurec et al. [16]). AC signals were produced by an Agilent 33210A 10 MHz Function/Arbitrary Waveform Generator and amplified using an Aetechron 7224 amplifier. The magnitude of the AC magnetic field was confirmed using a Wavecontrol SMP2 magnetometer. The coil used for in vivo experiments was custom‐built by the Bionterfaces lab, Department of Chemistry and Pharmacy at the University of Friedrich‐Alexander‐Universität Erlangen‐Nürnberg.

In all the experiments, a 28 mT sine wave at 5 Hz was applied for 20–30 s during the 120–180 s recording period. Stimulating timings for differentiated H9 stem cells were as follows: 0–30 s with no field, 30–60 s with AMF, 60–90 s with no field, 90–120 s with AMF, 120–150 s with no field, and 150–180 s with AMF. The stimulation protocol for hBSCs was as follows: 0–20 s with no field, 20–40 s with AMF, 40–60 s with no field, 60–80 s with AMF, 80–100 s with no field, and 100–120 s with AMF.

### 4.3 Ca^2+^ Transient Experiments

#### 4.3.1 Cell Culture

Cells were loaded with 1 µm Fluo‐4 AM dye (F14201, Invitrogen) and 1 µg/mL Propidium Iodide (P3566, Invitrogen) in phenol red‐free Hank's Balanced Salt Solution (HBSS;14025092, Gibco) for 30 min at 37°C. Following incubation, the cells were washed three times with HBSS, each lasting 5 min, by replacing half of the medium with fresh HBSS. After the final wash, half the HBSS was refreshed, and the cells designated for stimulation were incubated with MNDs for five additional minutes. Cells were then imaged using a Leica DIVI LFS microscope (Leica Biosystems, Wetzlar, Germany) with a 20x water‐dipping objective for 3 min. Changes in fluorescence (ΔF/F0) were calculated using the procedure described in Box 1 of Jia et al.

For the GsMTx4 condition, the cells were incubated with 1 µm GsMTx4 for 30 min post‐wash. MNDs were added during the final 5 min of incubation. Imaging was conducted in the same manner as that for the other conditions. For the Yoda1 condition, cells were treated similarly, but without the addition of MNDs. During imaging, 5 µm Yoda1 was added after 30 s to assess Piezo1 reactivity. A maximum of six recordings were used, and approximately 10 cells per recording were analyzed using the custom‐written Python script based on the work of Jia et al.

#### 4.3.2 Human Organotypic Brain Slice Cultures

All procedures involving human tissue were conducted with the approval of the Ethics Committee of Maastricht University (METC 2024‐0496). A day before the Ca^2+^ transient experiments, the tissue was separated to ensure that only one piece of tissue was in each transwell. On the morning of the experiments, the tissue was incubated with 2 µL of a 2 mg/mL MND solution. Grids were placed on top of the tissue and left to settle for a minimum of 1.5 h. Tissue was then loaded with a 1 µm Fluo‐4 AM dye (F14201, Invitrogen) in aCSF for 30 min at 37°C. After incubation with Fluo‐4, the tissue was gently washed for 5 min with HBSS. After washing the tissue, the insert was removed and placed in a 35 mm dish. The insert was then filled with fresh HBSS and imaged, as previously described.

### 4.4 Subjects

All procedures involving mice were conducted with the approval of the Animal Ethics Committee of Maastricht University (AVD10700202316737). The experiments were performed on 11 male naïve mice (C57BL/6J, Charles River), which were socially housed in a controlled environment maintained at 21°C ± 1°C with 40%–60% humidity, following a reversed 12:12 h light cycle (lights on at 21:00). All experimental manipulations were performed during the dark phase under red light, optimizing conditions for rodent activity. At the time of surgery, the mice were aged 10–12 weeks, and food and water were provided ad libitum. In the second cohort, 28 female wild‐type mice (C57BL/6J, Charles River) were housed under identical conditions.

### 4.5 Surgical Procedure

Before anesthesia, mice received a subcutaneous injection of buprenorphine at a dosage of 0.05 mg/kg as an analgesic. After 30 min, anesthesia was induced and maintained using isoflurane at concentrations of 4% for induction and 1.5%–3% for maintenance. Following adequate induction, each mouse was positioned in a stereotactic frame secured with ear bars and a mouse gas anesthesia head holder. Body temperature was monitored and maintained at 37°C using a thermoregulated heating pad. An ocular lubricant was applied to prevent drying of the eyes, and subcutaneous injection of 1% lidocaine was administered at the injection site for local anesthesia and analgesia.

Subsequently, a burr hole was drilled above the left STN at coordinates AP: −2.06 mm, ML: −1.50 mm, DV: −4.50 mm. A microinjection apparatus was used to inject 1.5 µL of a 1 mg/mL MNDs solution at an infusion rate of 100 nL/min. Following the injection, the syringe needle was left in the brain for an additional 5 min before being slowly removed. In the second cohort, 1.5 µL of a 1 mg/mL MNDs solution was injected into the left STN, and 0.2 µL of 6‐OHDA was unilaterally injected into the left medial forebrain bundle (AP: −1.2 mm, ML: −1.1 mm, DV: −5 mm) at a rate of 100 nL/ min (3 µg total). A total dropout rate of ∼10% was observed due to poor body condition of the 6‐OHDA mice.

### 4.6 In Vivo Magnetic Stimulation

All in vivo magnetic stimulations were conducted using a custom coil system, which allowed the mice to move freely during the stimulation period. The system was designed to generate a 28 mT, 5 Hz alternating current (AC) magnetic field aligned with the central axis of the animal chamber. This magnetic field was produced by a single coil with the animal chamber positioned at its center. A 1.32 mm‐thick copper wire was wound around a plastic coil frame comprising 990 turns. An Agilent 33210A 10 MHz Function/Arbitrary Waveform Generator provided a 5 Hz sine wave that was amplified using an Aetechron 7224 power amplifier. The output was connected to an AC coil, and the magnetic field strength was verified using a magnetometer (Wavecontrol SMP2). For all stimulation experiments, the mice were exposed to a magnetic field for 3 min, with the coil either turned on or off.

The quantitative relationship between AMF parameters and the resulting magnetomechanical torque exerted by vortex‐state MNDs, as well as magnetic field calibration and gradient analysis of the solenoid‐based stimulation system, were previously established and experimentally validated [16], and form the physical basis of the present study.

### 4.7 Behavioral Tests

All behavioral tests were performed under dim light conditions, and animals were allowed to acclimate to the behavioral room for a minimum of 1 h before the start of behavioral testing.

#### 4.7.1 Rotational Behavior

The circular arena was made of plexiglass (9 cm diameter and 30 cm height, fitting within the magnetic coil). The mice were placed into the cylinder, and the AMF stimulation was turned on or off for 3 min. Two researchers manually scored the videos independently, and rotations were classified as ipsilateral or contralateral around the body axis. The frequency was assessed per 3 min and per 30 sec time bins.

#### 4.7.2 Open Field Test (OFT)

The OFT consisted of a square arena with walls 25 cm high, measuring 40 cm in width and length. The behavior tracking system was operated in semi‐darkness for each mouse, employing a specialized tracking software (EthoVisionXT 15, Noldus Information Technology, Wageningen, The Netherlands). Each OFT lasted for 5 min, and each mouse was stimulated in an AMF coil for 3 min before testing.

#### 4.7.3 Rotarod

A rotarod with the ability to accelerate using a grooved rotating beam (3 cm) raised 16 cm above the platform (model 47650, Ugo Basile, Italy) was used to measure the coordination of the mice. The latency to fall of the rotating rod was recorded. The mice were tested in four sessions, each lasting 300 s, with and without AMF stimulation, prior to the start. Testing was performed at speeds starting at 4 rpm and accelerated to 40 rpm within 300 s. Between trials, mice were allowed at least 2 min of rest to reduce stress and fatigue. Values are expressed as the mean latency to fall from the rotarod in all test trials.

#### 4.7.4 Catwalk XT

The mice were assessed using the CatWalk‐automated gait analysis system (CatWalk XT 10.6, Noldus). The apparatus consisted of a long glass walkway illuminated by fluorescent light directed toward the side of the glass floor. In a dimly lit setting, the light is reflected downward, allowing a camera positioned beneath the glass to capture the footprints of the mouse as it moves along the walkway. The glass plate was cleaned and dried before testing each mouse to minimize the transmission of olfactory cues. In general, one successful test recording consisted of an average of four to five uninterrupted runs, with a comparable running speed and a maximum variation of 60%. The following parameters, including general, coordination, static, and dynamic aspects, were analyzed to assess individual paw function and overall gait patterns: run duration, number of steps, number of step patterns, step pattern regularity index (%), stride length, max contact area, print area, print length, print width, initial dual stance, terminal dual stance and body speed variation, three limb support and diagonal limb support.

### 4.8 Electrophysiological Recordings

Recordings were performed in one anaesthetized, head‐fixed, 6‐OHDA mouse. The mouse was preoperatively injected with ketamine‐medetomidine (K, 50 mg/kg; M, 0.2 mg/kg). Following adequate induction, the mouse was positioned in a custom‐built stereotactic frame secured with ear bars and a head holder. Body temperature was monitored and maintained at 37°C using a thermoregulated heating pad. Subsequently, a burr hole was drilled above the SNr at coordinates AP: −3.08 mm, ML: 1.5 mm, DV: 4.75 mm, and GPi at coordinates AP: −1.34 mm, ML: 1.75 mm, DV: 4.55 mm.

After the burr holes were drilled, the mouse was placed inside the AMF coil. The Neuropixels 1.0 probe was inserted inside the coil using a Sensapex uMP‐3 NP micromanipulator and slowly inserted into the brain until the desired target was reached. Once the target was reached, the tissue was left to settle for 10–15 min per target. Recordings were acquired in three blocks: pre‐stimulation, stimulation, and post‐stimulation. Neural signals were acquired for the AP band at a sampling rate of 30 kHz, band‐pass filtered between 0.3 and 10 kHz, and an LFP band at a sampling rate of 2.5 kHz, band‐pass filtered between 0.5 and 500 Hz.

The recordings were spike‐sorted using Kilosort4 and manually curated using Phy (https://github.com/cortex‐lab/phy) to identify single units. Single units were distinguished from multi‐units by imposing a threshold of 5% in the contamination parameter computed by Phy, which is roughly the event rate ratio in the central 2 ms of the clusters’ autocorrelogram. The data were then imported into Python 3.8.0 (Anaconda 23.3.1) and analyzed using custom‐written scripts adapted from SpikeInterface (https://spikeinterface.readthedocs.io). The mechanical stability of the recordings, that is, the lack of significant drifting, was verified by visual inspection of drift maps, and dorsal‐ventral positions of single units were aligned to the measured insertion depth of the probe.

Neurons with an average firing rate of <0.1 spikes/sec across the recording were excluded from the analysis. Spikes detected within 1 ms of stimulus cues were indissociable from the stimulation artefact and were therefore excluded from the study. To identify SNr neurons, we recorded a total of 13 single units from two separate recordings (*n* = 1), and to study GPi neurons, we recorded a total of 6 single units from two separate recordings (*n* = 1). Post‐recording histological analysis was conducted to verify probe placement and assess any tissue damage, ensuring that recordings were made from the intended cortical regions.

### 4.9 Tissue Collection

At the end of the experiments, the mice were euthanized with an overdose of pentobarbital. Transcardial perfusion was performed using Tyrode's buffer, followed by 4% PFA. Brains were then extracted and placed in fresh fixative for 24 h at 4°C. After fixation, the brains were transferred to 0.1% sodium azide (NaN_3_) at 4°C for long‐term storage. For sectioning, the brains were embedded in 10% porcine skin gelatin (Sigma–Aldrich, Zwijndrecht, The Netherlands) and cut into 30 µm coronal sections using a vibratome (Leica, Wetzlar, Germany). The sections were immediately placed in 0.1% NaN_3_ and stored at 4°C.

### 4.10 Immunohistochemistry

#### 4.10.1 Mouse Brains

For immunohistochemistry, the sections were incubated overnight with polyclonal rabbit anti‐c‐Fos primary antibody (1:1000, Abcam, ab190289). The tissue was incubated with biotinylated donkey anti‐rabbit secondary antibody (1:400, Invitrogen, Carlsbad, CA, USA) and avidin‐biotin‐peroxidase complex (1:800, Elite ABC kit, Vectastain, Burlingame, CA, USA). The staining was visualized using 3,3′‐diaminobenzidine (DAB) combined with NiCl2 intensification. The slides were dehydrated and coverslipped using Entellan.

For immunofluorescence, the sections were incubated overnight with either polyclonal sheep anti‐TH (1:2000, Abcam, ab113), polyclonal rabbit anti‐IBA1 (1:2000, Invitrogen, PA5‐27436), or biotin‐conjugated rabbit anti‐GFAP (1:2000, Merck, MAB3402B) primary antibody. Staining was visualized by immunofluorescence with donkey anti‐sheep Alexa 488 (1:200), donkey anti‐rabbit Alexa 594 (1:200), or streptavidin 594 (1:1000).

#### 4.10.2 Human Organotypic Brain Slice Cultures

##### 4.10.2.1 C‐Fos Expression

For immunohistochemistry, the sections were incubated overnight with polyclonal rabbit anti‐c‐Fos primary antibody (1:1000, ab190289, Abcam). c‐Fos immunohistochemistry included biotinylated donkey anti‐rabbit secondary antibody (1:200, Invitrogen, Carlsbad, CA, USA) and avidin‐biotin‐peroxidase complex (1:400, Elite ABC kit, Vectastain, Burlingame, CA, USA). The staining was visualized using 3,3′‐diaminobenzidine (DAB) combined with NiCl_2_ intensification.

##### 4.10.2.2 TRPV4 and Piezo1 Expression

For Immunofluorescence, hBSC sections were incubated overnight with polyclonal rabbit anti‐TRPV4 (1:500, Alamone Labs, ACC‐034) or for four nights with polyclonal rabbit anti‐Piezo1 (1:100, Novus Biologicals, NBP1‐78537) and monoclonal mouse anti‐NeuN primary antibody (1:100, EMD Millipore, MAB377). TRPV4 and Piezo1 were visualized with donkey anti‐rabbit Alexa 488 (1:200, Invitrogen, Carlsbad, CA, USA), and NeuN was visualized using donkey anti‐mouse Alexa 594 (1:200, Invitrogen, Carlsbad, CA, USA). The nuclei were stained with Hoechst (1:10.000). Photomicrographs were taken using an Olympus Disk Spinning Unit (DSU) with a 20x objective.

#### 4.10.3 Stereology

The number of c‐Fos‐, TH‐, IBA1‐, and GFAP‐positive cells was counted using the stereological procedure, optical fractionator. Counts were performed using a microscope (Olympus BX51W1), a motorized stage, and StereoInvestigator software (MicroBrightField, Williston, VT). All positive cells in an average of two to three sections per region of interest, 300 µm apart, were counted using a 20× objective. The total number of positive cells was estimated as a function of the number of cells counted and sampling probability.

The number of c‐Fos‐positive cells in hBSC tissue was counted using the same stereological procedure. All positively stained cells in an average of three sections were counted using a 20× objective. The total number of positive cells was estimated as a function of the number of cells counted and sampling probability.

#### 4.10.4 Immunocytochemistry

The cells were seeded at 2.0 × 105 cells per well. Once the cells reached 80% confluence, the culture medium was removed, and the cells were washed with PBS. The cells were then fixed with 4% paraformaldehyde for 10 min. The cells were then washed again, incubated with 3% Triton‐X for 10 min, and blocked for 30 min in 5% normal goat serum. The fixed cells were incubated with polyclonal anti‐Piezo1 and polyclonal anti‐TRPV4 (1:200; Alomone Labs, Israel) primary antibodies for 50 min at room temperature. Next, the cells were incubated with donkey anti‐rabbit Alexa 488 for Piezo1 or donkey anti‐rabbit Alexa 594 for TRPV4 (1:200) secondary antibodies for 50 min at room temperature.

### 4.11 Statistical Analysis

All data are represented as mean ± standard error of the mean (S.E.M.). Statistical analyses were performed using GraphPad Prism software (version 10). Data normality and homogeneity of variance were checked using the Shapiro–Wilk test and normality plots. All behavioral data from the naïve experiment were analyzed using a dependent one‐tailed *t*‐test to compare before and after AMF stimulation. Stereological cell counts were analyzed using a dependent samples *t*‐test for IBA1 and GFAP, an independent samples *t*‐test for TH, and a one‐way ANOVA for c‐Fos. All behavioral data from 6‐OHDA experiments were analyzed using a one‐tailed independent *t*‐test. Electrophysiology data were analyzed using repeated‐measures ANOVA with post hoc Bonferroni correction. Statistical significance was set at *p* < 0.05.

## Funding

A.W. and S.H. acknowledge that this publication is part of the project “Minimally‐Invasive Neural Devices with Magnetic Nanodiscs for Advanced Precision– MINDMAP” with file number OCENW.M.22.436 of the research program NWO Open Competition. D.G. acknowledges the funding from EIC Pathfinder Open BRAINSTORM (GA101099355) and the ERC Starting Grant 2023 BRAINMASTER (GA101116410).

## Conflicts of Interest

The authors declare no conflicts of interest.

## Supporting information




**Supporting File**: advs75097‐sup‐0001‐SuppMat.docx

## Data Availability

The data that support the findings of this study are available from the corresponding author upon reasonable request.

## References

[1] M. K. Lyons , ed., Deep Brain Stimulation: Current and Future Clinical Applications. (Elsevier, 2011). 10.4065/mcp.2011.0045 PMC3127561 21646303

[2] E. Kocabicak , Y. Temel , A. Höllig , B. Falkenburger , and S. K. Tan , “Current Perspectives on Deep Brain Stimulation for Severe Neurological and Psychiatric Disorders,” Neuropsychiatric Disease and Treatment 11 (2015): 1051–1066. 25914538 10.2147/NDT.S46583 PMC4399519

[3] M. Lange , J. Mauerer , J. Schlaier , et al., “Underutilization of Deep Brain Stimulation for Parkinson's Disease? A Survey on Possible Clinical Reasons,” Acta Neurochirurgica 159, no. 5 (2017): 771–778, 10.1007/s00701-017-3122-3. 28258308

[4] A. M. Lozano , N. Lipsman , H. Bergman , et al., “Deep Brain Stimulation: Current Challenges and Future Directions,” Nature Reviews Neurology 15, no. 3 (2019): 148–160, 10.1038/s41582-018-0128-2. 30683913 PMC6397644

[5] Y. Temel and A. Jahanshahi , “Treating Brain Disorders With Neuromodulation,” Science 347, no. 6229 (2015): 1418–1419, 10.1126/science.aaa9610. 25814569

[6] J. Rivnay , H. Wang , L. Fenno , K. Deisseroth , and G. G. Malliaras , “Next‐generation Probes, Particles, and Proteins for Neural Interfacing,” Science Advances 3, no. 6 (2017): 1601649, 10.1126/sciadv.1601649. PMC5466371 28630894

[7] L. Signorelli , S.‐A. Hescham , A. Pralle , and D. Gregurec , “Magnetic Nanomaterials for Wireless Thermal and Mechanical Neuromodulation,” Iscience 25 (2022): 105401. 36388996 10.1016/j.isci.2022.105401 PMC9641224

[8] K. L. Kozielski , A. Jahanshahi , H. B. Gilbert , et al., “Nonresonant Powering of Injectable Nanoelectrodes Enables Wireless Deep Brain Stimulation in Freely Moving Mice,” Science Advances 7, no. 3 (2021): abc4189, 10.1126/sciadv.abc4189. PMC7806222 33523872

[9] Y. J. Kim , N. Kent , E. Vargas Paniagua , et al., “Magnetoelectric Nanodiscs Enable Wireless Transgene‐free Neuromodulation,” Nature Nanotechnology 20, no. 1 (2025): 121–131, 10.1038/s41565-024-01798-9. PMC11750723 39394431

[10] S. S. Tower , “Arthroprosthetic Cobaltism: Neurological and Cardiac Manifestations in Two Patients With Metal‐on‐Metal Arthroplasty,” Journal of Bone and Joint Surgery 92, no. 17 (2010): 2847–2851, 10.2106/JBJS.J.00125. 21037026

[11] T. Ikeda , K. Takahashi , T. Kabata , D. Sakagoshi , K. Tomita , and M. Yamada , “Polyneuropathy Caused by Cobalt–Chromium Metallosis After Total Hip Replacement,” Muscle & nerve 42, no. 1 (2010): 140–143, 10.1002/mus.21638. 20544916

[12] S.‐A. Hescham , P.‐H. Chiang , D. Gregurec , et al., “Magnetothermal Nanoparticle Technology Alleviates Parkinsonian‐Like Symptoms in Mice,” Nature Communications 12, no. 1 (2021): 5569, 10.1038/s41467-021-25837-4. PMC8458499 34552093

[13] R. Chen , G. Romero , M. G. Christiansen , A. Mohr , and P. Anikeeva , “Wireless Magnetothermal Deep Brain Stimulation,” Science 347, no. 6229 (2015): 1477–1480, 10.1126/science.1261821. 25765068

[14] C.‐L. Su , C.‐C. Cheng , P.‐H. Yen , J.‐X. Huang , Y.‐J. Ting , and P.‐H. Chiang , “Wireless Neuromodulation in Vitro and in Vivo by Intrinsic TRPC‐mediated Magnetomechanical Stimulation,” Communications Biology 5, no. 1 (2022): 1166, 10.1038/s42003-022-04124-y. 36323817 PMC9630493

[15] M. Alipour , M. Abdolmaleki , Y. Shabanpour , et al., “Advances in Magnetic Field Approaches for Non‐Invasive Targeting Neuromodulation,” Frontiers in Human Neuroscience 19 (2025): 1489940, 10.3389/fnhum.2025.1489940. 40356879 PMC12066545

[16] D. Gregurec , A. W. Senko , A. Chuvilin , et al., “Magnetic Vortex Nanodiscs Enable Remote Magnetomechanical Neural Stimulation,” ACS nano 14, no. 7 (2020): 8036–8045, 10.1021/acsnano.0c00562. 32559057 PMC8592276

[17] F. Zhang , H. Mehta , H. H. Choudhary , R. Islam , and K. A. Hanafy , “TRPV4 Channel in Neurological Disease: From Molecular Mechanisms to Therapeutic Potential,” Molecular Neurobiology 62 (2025): 3877–3891. 39333347 10.1007/s12035-024-04518-5 PMC11790740

[18] L. Soattin , M. Fiore , P. Gavazzo , et al., “The Biophysics of piezo1 and piezo2 Mechanosensitive Channels,” Biophysical chemistry 208 (2016): 26–33, 10.1016/j.bpc.2015.06.013. 26259784

[19] C.‐L. Su , P.‐H. Yen , C.‐C. Cheng , and P.‐H. Chiang , “Remote Deep Brain Stimulation by Transgene‐Free Magnetomechanical Approach,” bioRxiv 5 (2022): 1166.

[20] J.‐U. Lee , W. Shin , Y. Lim , et al., “Non‐Contact Long‐range Magnetic Stimulation of Mechanosensitive Ion Channels in Freely Moving Animals,” Nature Materials 20, no. 7 (2021): 1029–1036, 10.1038/s41563-020-00896-y. 33510447

[21] Q. Zheng , H. Liu , W. Yu , et al., “Mechanical Properties of the Brain: Focus on the Essential Role of Piezo1‐Mediated Mechanotransduction in the CNS,” Brain and Behavior 13, no. 9 (2023): 3136, 10.1002/brb3.3136. PMC10498085 37366640

[22] H. Kumar , S.‐H. Lee , K.‐T. Kim , X. Zeng , and I. Han , “TRPV4: A Sensor for Homeostasis and Pathological Events in the CNS,” Molecular Neurobiology 55, no. 11 (2018): 8695–8708, 10.1007/s12035-018-0998-8. 29582401

[23] R. Syeda , “Piezo1 Channels Are Inherently Mechanosensitive,” Biophysical Journal 112, no. 3 (2017): 8A, 10.1016/j.bpj.2016.11.070. PMC5129625 27829145

[24] J. P. White , M. Cibelli , L. Urban , B. Nilius , J. G. McGeown , and I. Nagy , “TRPV4: Molecular Conductor of a Diverse Orchestra,” Physiological Reviews 96, no. 3 (2016): 911–973, 10.1152/physrev.00016.2015. 27252279

[25] J. Li , B. Hou , S. Tumova , et al., “Piezo1 integration of Vascular Architecture With Physiological Force,” Nature 515, no. 7526 (2014): 279–282, 10.1038/nature13701. 25119035 PMC4230887

[26] H. Jäntti , V. Sitnikova , Y. Ishchenko , et al., “Microglial Amyloid Beta Clearance Is Driven by PIEZO1 Channels,” Journal of Neuroinflammation 19, no. 1 (2022): 147, 10.1186/s12974-022-02486-y. 35706029 PMC9199162

[27] T. M. Suchyna , “Piezo Channels and GsMTx4: Two Milestones in Our Understanding of Excitatory Mechanosensitive Channels and Their Role in Pathology,” Progress in Biophysics and Molecular Biology 130 (2017): 244–253, 10.1016/j.pbiomolbio.2017.07.011. 28778608 PMC5716857

[28] M. A. Spassova , T. Hewavitharana , W. Xu , J. Soboloff , and D. L. Gill , “A Common Mechanism Underlies Stretch Activation and Receptor Activation of TRPC6 Channels,” Proceedings of the National Academy of Sciences 103, no. 44 (2006): 16586–16591, 10.1073/pnas.0606894103. PMC1637625 17056714

[29] C. Bae , F. Sachs , and P. A. Gottlieb , “The Mechanosensitive Ion Channel Piezo1 Is Inhibited by the Peptide GsMTx4,” Biochemistry 50, no. 29 (2011): 6295–6300, 10.1021/bi200770q. 21696149 PMC3169095

[30] P. Calabresi , B. Picconi , A. Tozzi , V. Ghiglieri , and M. Di Filippo , “Direct and Indirect Pathways of Basal Ganglia: A Critical Reappraisal,” Nature neuroscience 17, no. 8 (2014): 1022–1030, 10.1038/nn.3743. 25065439

[31] J. I. Morgan and T. Curran , “Role of Ion Flux in the Control of c‐fos Expression,” Nature 322, no. 6079 (1986): 552–555, 10.1038/322552a0. 2426600

[32] D. J. Lee , C. S. Lozano , R. F. Dallapiazza , and A. M. Lozano , “Current and Future Directions of Deep Brain Stimulation for Neurological and Psychiatric Disorders,” Journal of neurosurgery 131, no. 2 (2019): 333–342, 10.3171/2019.4.JNS181761. 31370011

[33] E. A. Kappos , P. K. Sieber , P. E. Engels , et al., “Validity and Reliability of the CatWalk System as a Static and Dynamic Gait Analysis Tool for the Assessment of Functional Nerve Recovery in Small Animal Models,” Brain and behavior 7, no. 7 (2017): 00723, 10.1002/brb3.723. PMC5516599 28729931

[34] S. E. Park , K.‐I. Song , H. Kim , S. Chung , and I. Youn , “Graded 6‐OHDA‐induced Dopamine Depletion in the Nigrostriatal Pathway Evokes Progressive Pathological Neuronal Activities in the Subthalamic Nucleus of a Hemi‐Parkinsonian Mouse,” Behavioural Brain Research 344 (2018): 42–47, 10.1016/j.bbr.2018.02.014. 29452192

[35] G. C. McConnell , R. Q. So , J. D. Hilliard , P. Lopomo , and W. M. Grill , “Effective Deep Brain Stimulation Suppresses Low‐Frequency Network Oscillations in the Basal Ganglia by Regularizing Neural Firing Patterns,” The Journal of Neuroscience 32, no. 45 (2012): 15657–15668, 10.1523/JNEUROSCI.2824-12.2012. 23136407 PMC3502634

[36] B. D. Swan , D. T. Brocker , J. D. Hilliard , et al., “Short Pauses in Thalamic Deep Brain Stimulation Promote Tremor and Neuronal Bursting,” Clinical Neurophysiology 127, no. 2 (2016): 1551–1559, 10.1016/j.clinph.2015.07.034. 26330131 PMC4747847

[37] Z.‐G. Ni , R. Bouali‐Benazzouz , D.‐M. Gao , A.‐L. Benabid , and A. Benazzouz , “Time‐Course of Changes in Firing Rates and Firing Patterns of Subthalamic Nucleus Neuronal Activity After 6‐OHDA‐Induced Dopamine Depletion in Rats,” Brain Research 899, no. 1‐2 (2001): 142–147, 10.1016/S0006-8993(01)02219-3. 11311875

[38] C.‐H. Tai , “Subthalamic Burst Firing: A Pathophysiological Target in Parkinson's Disease,” Neuroscience & Biobehavioral Reviews 132 (2022): 410–419, 10.1016/j.neubiorev.2021.11.044. 34856222

[39] L. Perestelo‐Pérez , A. Rivero‐Santana , J. Pérez‐Ramos , P. Serrano‐Pérez , J. Panetta , and P. Hilarion , “Deep Brain Stimulation in Parkinson's Disease: Meta‐analysis of Randomized Controlled Trials,” Journal of Neurology 261 (2014): 2051–2060, 10.1007/s00415-014-7254-6. 24487826

[40] C. Hamani , J. A. Saint‐Cyr , J. Fraser , M. Kaplitt , and A. M. Lozano , “The Subthalamic Nucleus in the Context of Movement Disorders,” Brain 127, no. 1 (2004): 4–20, 10.1093/brain/awh029. 14607789

[41] C. Hamani , G. Florence , H. Heinsen , et al., “Subthalamic Nucleus Deep Brain Stimulation: Basic Concepts and Novel Perspectives,” Eneuro 4, no. 5 (2017), 10.1523/ENEURO.0140-17.2017. PMC5617209 28966978

[42] J. D. Rolston , D. J. Englot , P. A. Starr , and P. S. Larson , “An Unexpectedly High Rate of Revisions and Removals in Deep Brain Stimulation Surgery: Analysis of Multiple Databases,” Parkinsonism & Related Disorders 33 (2016): 72–77, 10.1016/j.parkreldis.2016.09.014. 27645504 PMC5240785

[43] M.‐R. Kim , J. Y. Yun , B. Jeon , et al., “Patients' Reluctance to Undergo Deep Brain Stimulation for Parkinson's Disease,” Parkinsonism & related disorders 23 (2016): 91–94, 10.1016/j.parkreldis.2015.11.010. 26686260

[44] J. Liu , R. Munshi , M. He , S. D. Parker , and A. Pralle , “Deep Brain Magnetothermal Silencing of Dopaminergic Neurons via Endogenous TREK1 Channels Abolishes Place Preference in Mice,” bioRxiv (2022), 10.1101/2022.04.12.487994.

[45] R. Munshi , S. M. Qadri , Q. Zhang , I. Castellanos Rubio , P. Del Pino , and A. Pralle , “Magnetothermal Genetic Deep Brain Stimulation of Motor Behaviors in Awake, Freely Moving Mice,” Elife 6 (2017): 27069, 10.7554/eLife.27069. PMC5779110 28826470

[46] W. Liedtke and J. M. Friedman , “Abnormal Osmotic Regulation in trpv4‐/‐mice,” Proceedings of the National Academy of Sciences 100, no. 23 (2003): 13698–13703, 10.1073/pnas.1735416100. PMC263876 14581612

[47] A. Nambu , H. Tokuno , and M. Takada , “Functional Significance of the Cortico–Subthalamo–Pallidal ‘Hyperdirect’ Pathway,” Neuroscience Research 43, no. 2 (2002): 111–117, 10.1016/S0168-0102(02)00027-5. 12067746

[48] C. Bosch‐Bouju , B. I. Hyland , and L. C. Parr‐Brownlie , “Motor Thalamus Integration of Cortical, Cerebellar and Basal Ganglia Information: Implications for Normal and Parkinsonian Conditions,” Frontiers in Computational Neuroscience 7 (2013): 163, 10.3389/fncom.2013.00163. 24273509 PMC3822295

[49] S. Arber and R. M. Costa , “Networking Brainstem and Basal Ganglia Circuits for Movement,” Nature Reviews Neuroscience 23, no. 6 (2022): 342–360, 10.1038/s41583-022-00581-w. 35422525

[50] L.‐H. Shi , D. J. Woodward , F. Luo , K. Anstrom , T. Schallert , and J.‐Y. Chang , “High‐frequency Stimulation of the Subthalamic Nucleus Reverses Limb‐use Asymmetry in Rats With Unilateral 6‐hydroxydopamine Lesions,” Brain Research 1013, no. 1 (2004): 98–106, 10.1016/j.brainres.2004.03.053. 15196972

[51] J.‐Y. Chang , L.‐H. Shi , F. Luo , and D. J. Woodward , “High Frequency Stimulation of the Subthalamic Nucleus Improves Treadmill Locomotion in Unilateral 6‐hydroxydopamine Lesioned Rats,” Brain Research 983, no. 1‐2 (2003): 174–184, 10.1016/S0006-8993(03)03053-1. 12914978

[52] X. Fang , K. Sugiyama , S. Akamine , W. Sun , and H. Namba , “The Different Performance Among Motor Tasks During the Increasing Current Intensity of Deep Brain Stimulation of the Subthalamic Nucleus in Rats With Different Degrees of the Unilateral Striatal Lesion,” Neuroscience letters 480, no. 1 (2010): 64–68, 10.1016/j.neulet.2010.06.004. 20573573

[53] R. Vlamings , V. Visser‐Vandewalle , G. Koopmans , et al., “High Frequency Stimulation of the Subthalamic Nucleus Improves Speed of Locomotion but Impairs Forelimb Movement in Parkinsonian Rats,” Neuroscience 148, no. 3 (2007): 815–823, 10.1016/j.neuroscience.2007.06.043. 17706885

[54] C. Xiao , J. Y‐w , L. Y‐w , T. Jia , C. Yin , and Z. C‐y , “Differential Modulation of Subthalamic Projection Neurons by Short‐term and Long‐term Electrical Stimulation in Physiological and Parkinsonian Conditions,” Acta Pharmacologica Sinica 43, no. 8 (2022): 1928–1939, 10.1038/s41401-021-00811-4. 34880404 PMC9343451

[55] C. de Hemptinne , N. C. Swann , J. L. Ostrem , et al., “Therapeutic Deep Brain Stimulation Reduces Cortical Phase‐Amplitude Coupling in Parkinson's Disease,” Nature neuroscience 18, no. 5 (2015): 779–786, 10.1038/nn.3997. 25867121 PMC4414895

[56] E. Faggiani and A. Benazzouz , “Deep Brain Stimulation of the Subthalamic Nucleus in Parkinson's Disease: From History to the Interaction With the Monoaminergic Systems,” Progress in Neurobiology 151 (2017): 139–156, 10.1016/j.pneurobio.2016.07.003. 27412110

[57] J. Kefauver , A. Ward , and A. Patapoutian , “Discoveries in Structure and Physiology of Mechanically Activated Ion Channels,” Nature 587, no. 7835 (2020): 567–576, 10.1038/s41586-020-2933-1. 33239794 PMC8477435

[58] B. Coste , S. E. Murthy , J. Mathur , et al., “Piezo1 ion Channel Pore Properties Are Dictated by C‐terminal Region,” Nature Communications 6, no. 1 (2015): 7223, 10.1038/ncomms8223. PMC4445471 26008989

[59] M. C. Marchetto , H. Belinson , Y. Tian , et al., “Altered Proliferation and Networks in Neural Cells Derived From Idiopathic Autistic Individuals,” Molecular Psychiatry 22, no. 6 (2017): 820–835, 10.1038/mp.2016.95. 27378147 PMC5215991

[60] A. Bak , H. Koch , K. M. J. van Loo , et al., “Human Organotypic Brain Slice Cultures: A Detailed and Improved Protocol for Preparation and Long‐term Maintenance,” Journal of Neuroscience Methods 404 (2024): 110055, 10.1016/j.jneumeth.2023.110055. 38184112

[61] H. Jia , N. L. Rochefort , X. Chen , and A. Konnerth , “In Vivo Two‐photon Imaging of Sensory‐Evoked Dendritic Calcium Signals in Cortical Neurons,” Nature protocols 6, no. 1 (2011): 28–35, 10.1038/nprot.2010.169. 21212780

[62] M. Pachitariu , S. Sridhar , J. Pennington , and C. Stringer , “Spike Sorting With Kilosort4,” Nature Methods 21, no. 5 (2024): 914–921, 10.1038/s41592-024-02232-7. 38589517 PMC11093732

