# Supplementary material for: Remote Magnetomechanical Neuromodulation Uncovers Therapeutic Mechanisms for Alleviating Parkinsonian Symptoms in Freely Moving Mice
Source: Adv Sci (Weinh). 2026 Apr 3:e75097. Online ahead of print. doi: 10.1002/advs.75097 (PMC13325837; doi:10.1002/advs.75097)
Supplement: Supplementary file 1 — Supporting File: advs75097‐sup‐0001‐SuppMat.docx [file ADVS-9999-e75097-s001.docx]

**Supplementary Information**

***
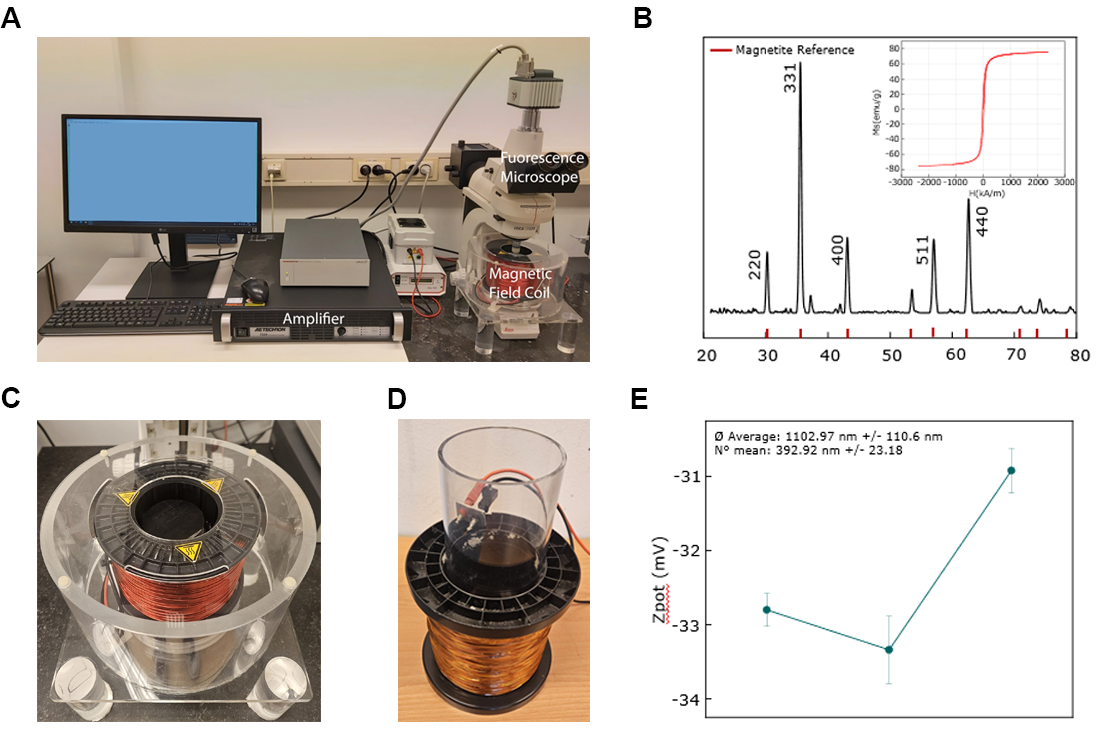
***

***Figure S1. Magnetomechanical setup and MNDs characterisation. A.*** *Overview of in vitro and ex vivo magnetomechanical stimulation setup.* ***B.*** *XRD spectrum and VSM analysis of the MNDs confirm the crystalline phase and magnetic saturation of magnetite.* ***C.*** *Overview of in vivo magnetomechanical stimulation setup.* ***D.*** *Zeta potential and average diameter of MNDs after PMAO coating.*

***
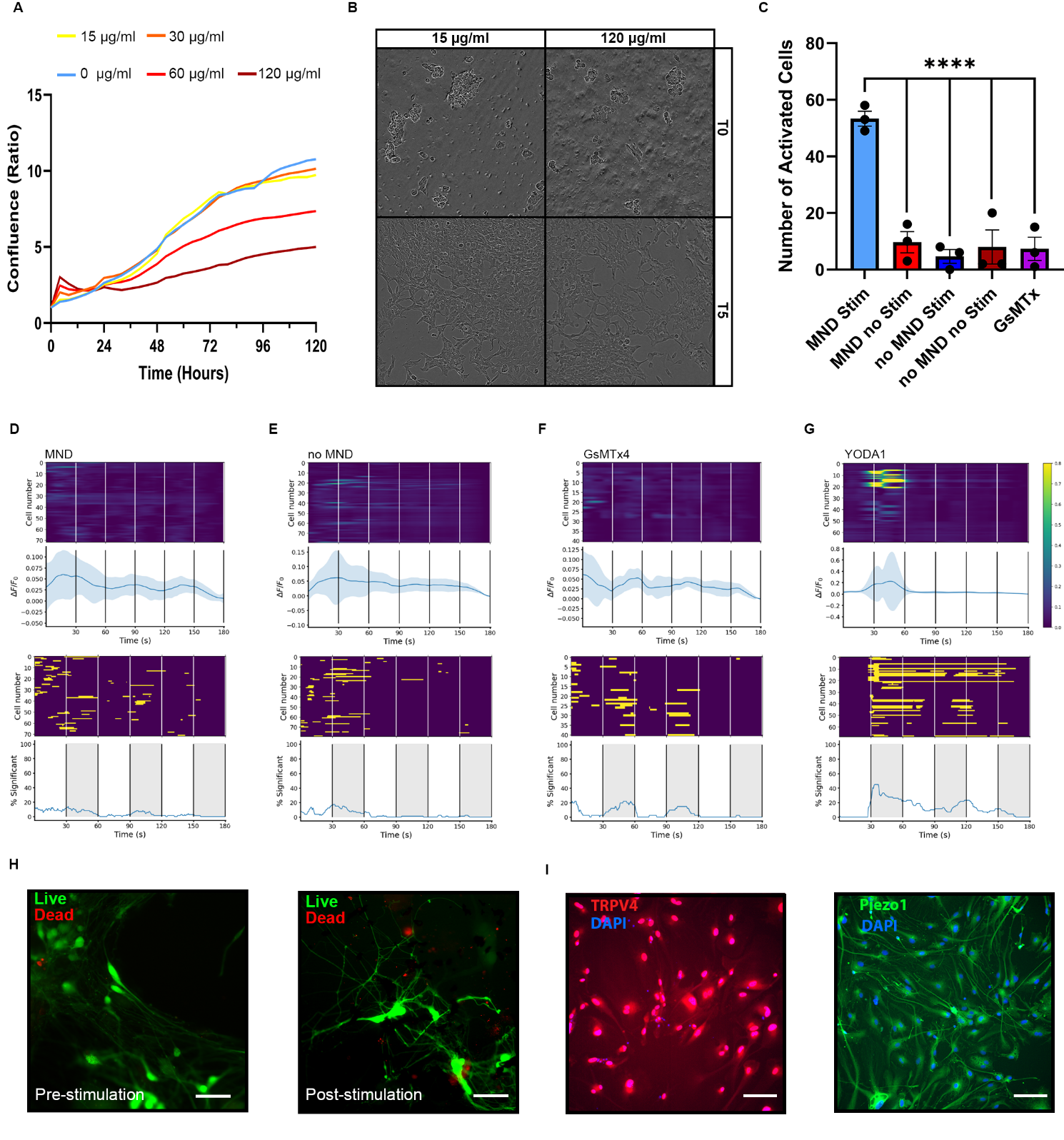
*Figure S2. In vitro magnetomechanical control. A.** Real-time quantification of cell death in HEK293 cells following 5 days of exposure to MNDs at various concentrations was performed in triplicate. The concentration in all further in vitro experiments was 30 µg/ml. **B.** Images of cells immediately after exposure and 5 days of exposure to MNDs at 15 µg/ml and 120 µg/ml. Scale bar = 150 µm. **C.** Maximum number of cells responded, averaged across the three stimulation epochs. **D-E**Heatmaps (top) showing fluorescence changes expressed as ΔF/F_0_ and significance plots (bottom) indicating the presence of absence of statistically significant response relative to baseline recorded from Fluo-4 AM transients observed in Piezo1 and TRPV4 expressing hESC-derived neurons during magnetic field stimulus with (C) and without (D) MNDs during AMF Off. **F.** Magnetomechanical cation channel inhibition of Piezo1 and TRPV4 through GsMTx4. **G.** Pharmacological activation of Piezo1 through YODA1. Each condition was repeated across six different cultures, with ~10 cells randomly selected for analysis. **H.** Confocal images of hESC-derived neurons loaded with the calcium indicator Fluo-4 AM and propidium iodide (PI) for live/dead staining before and after mechanostimulation. Fluo-4 AM stains live cells in green, and PI stains dead cells in red. Scale bar = 50 µm. **I.** Confocal microscope images of hESC-derived neurons stained for TRPV4 (red), Piezo1 (green) and DAPI (blue). Scale bar = 50 µm. **** Indicates p < 0.0001, Repeated measures one-way ANOVA, data presented as mean ± S.E.M.

***
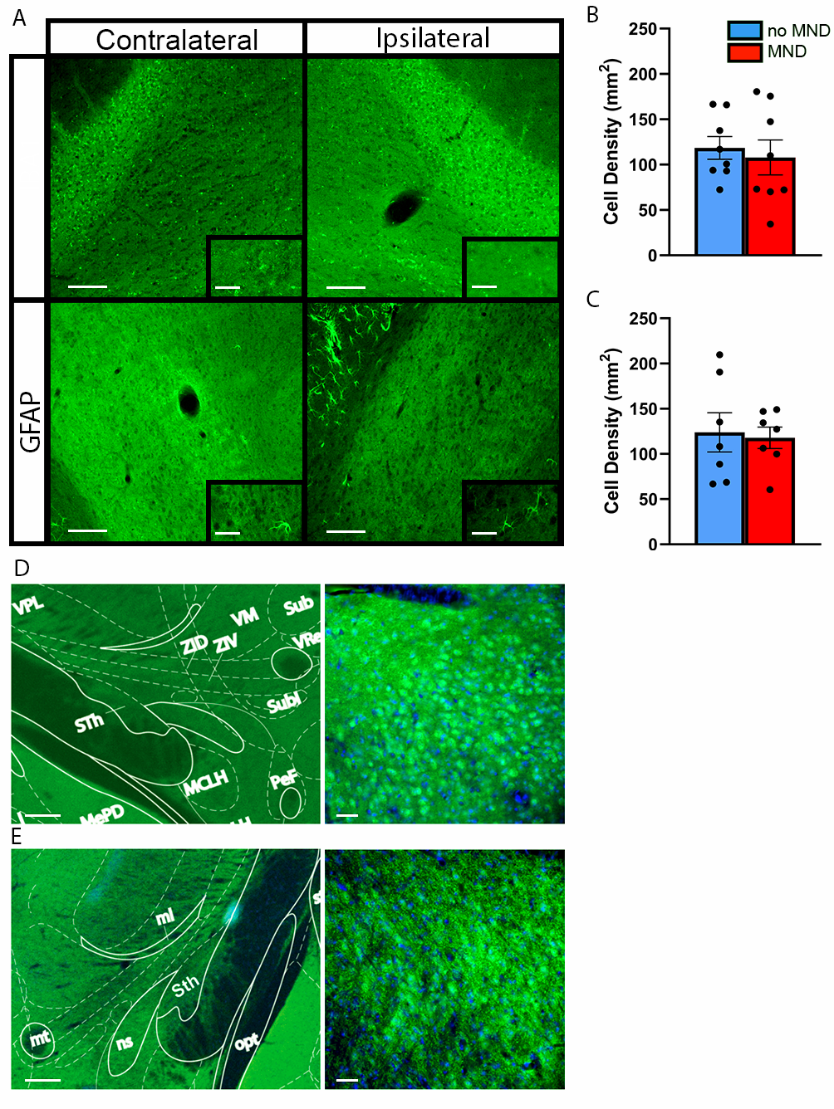
***

**Figure S3. Glial response following MNDs exposure. A.** Representative low- *(200 µm) and high-power (50 µm) photomicrographs of Allograft inflammatory factor 1 (IBA1)- and Glial fibrillary acidic protein (GFAP)-positive cells in the STN contralateral and ipsilateral to the MND injection.* ***B.*** *MNDs exposure showed no increase in IBA1-positive cells in the STN.* ***C.*** *MNDs exposure showed no increase in GFAP-positive cells in the STN.* ***D.*** *Coronal mouse brain section with atlas overlay showing Piezo1 (green) and DAPI (blue) (scale bar =100 µm) and corresponding high-power photomicrograph (scale bar = 50 µm).* ***E.*** *Coronal mouse brain section with atlas overlay showing TRPV4 (green) and DAPI (blue) (scale bar = 100 µm) and corresponding high-power photomicrograph (scale bar = 50 µm). One-tailed dependent t-test, data presented as mean* ± S.E.M.

*
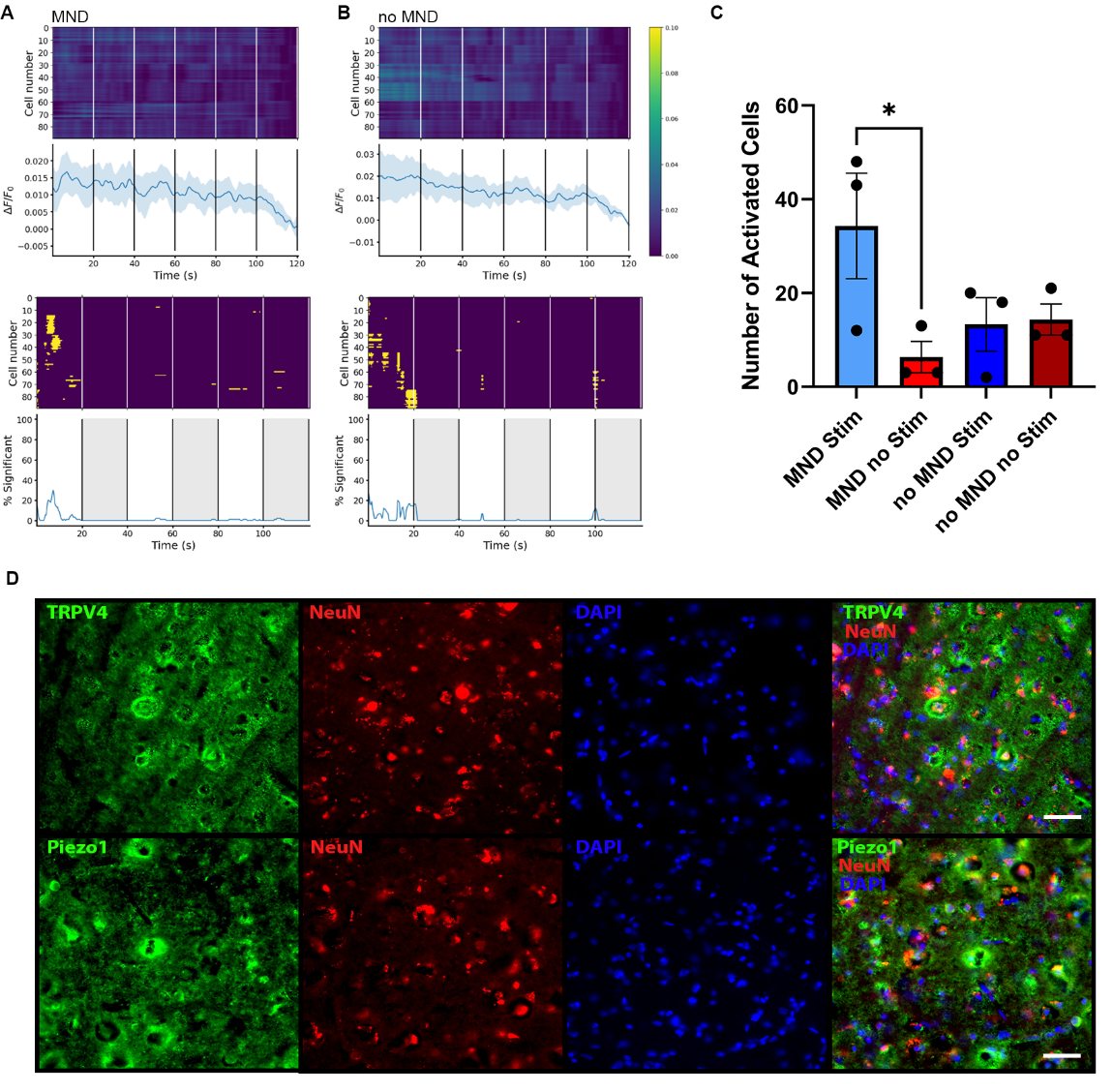
*

**Figure S4. Ex vivo magnetomechanical control.** Heatmaps (top) and significance plots (bottom) of fluorescence intensity changes recorded from Fluo-4 AM transients observed in Piezo1 and TRPV4 expressing hBSC during magnetic field stimulus with **(A)** and without **(B)** MNDs during AMF Off. Each condition was repeated across six different cultures, with ~15 cells randomly selected for analysis. **C.** Maximum number of cells responded, averaged across the three stimulation epochs. **D.** Representative high-power photomicrographs of hBSC stained for NeuN (red) and TRPV4 or Piezo1 (green) and DAPI (blue) (scale bar = 50 µm). * Indicates p < 0.05, Repeated measures one-way ANOVA, data presented as mean ± S.E.M.


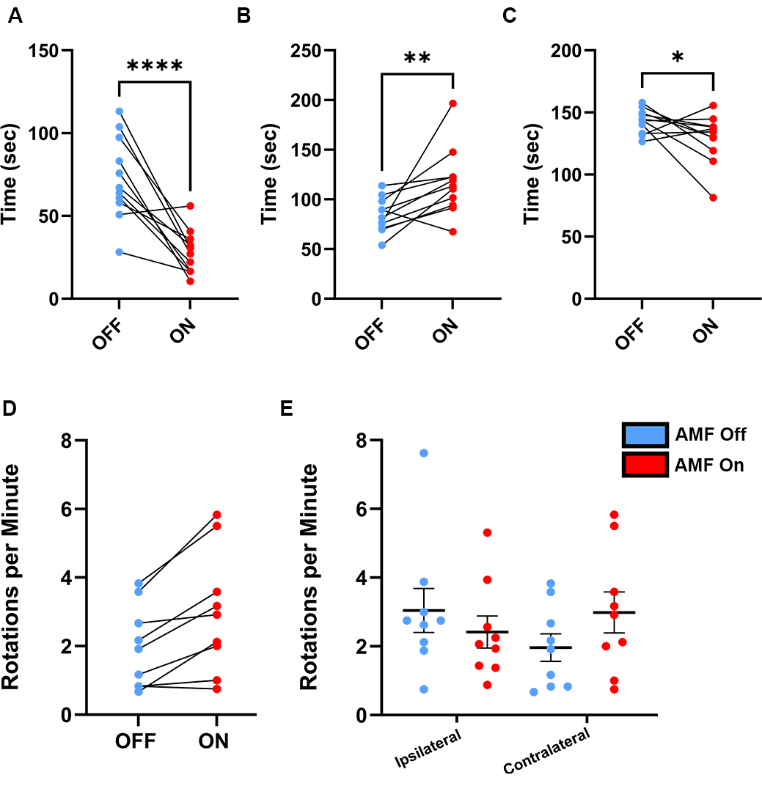


**Figure S5. Remote magnetomechanical neuromodulation of mouse behaviour. A.** Decreased total time spent in the centre (p=0.0002). **B.** Increased total time spent in the corners (p=0.0059). **C.** Decreased total time spent at the walls (p=0.0407) during the 5-minute OFT. **D.** Contralateral rotations of STN mDBS (n=11) during AMF On and Off conditions. **E.** Rotational behaviour presented in ipsilateral and contralateral rotations of STN mDBS (n=11) during AMF On and Off. * Indicates p < 0.05, ** < 0.01, **** < 0.0001. One-tailed t-test, data presented as mean ± S.E.M.


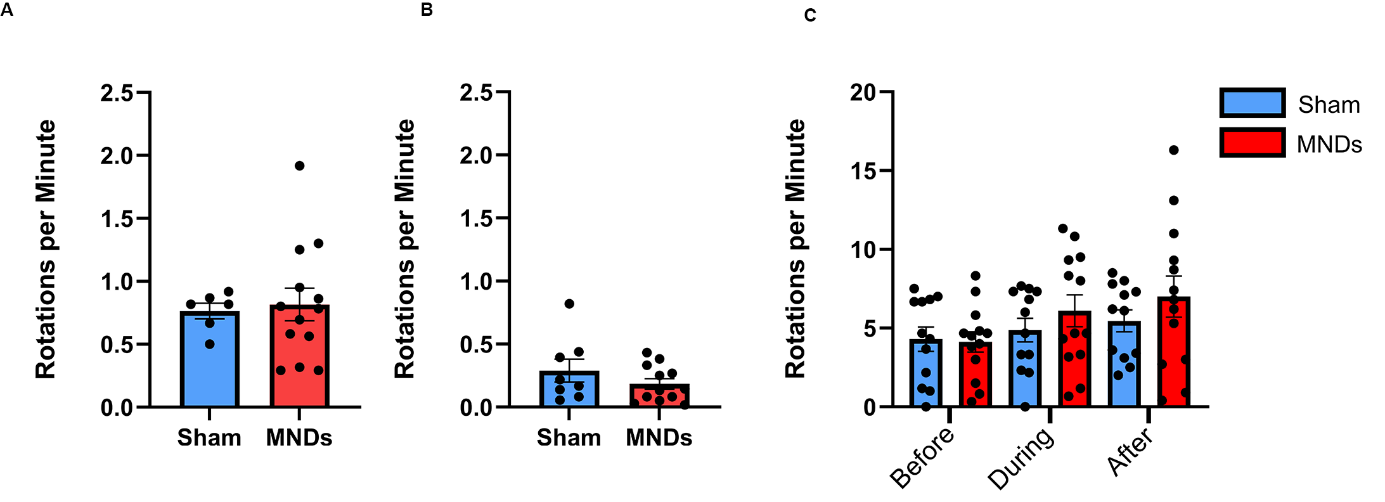


**Figure S6.** **The effects of magnetomechanical STN DBS on rotational behaviour. A.** Ipsilateral rotations per minute. **B.** Contralateral rotations per minute of the sham (n=8) and STN mDBS (n=13). Data presented as mean ± S.E.M.


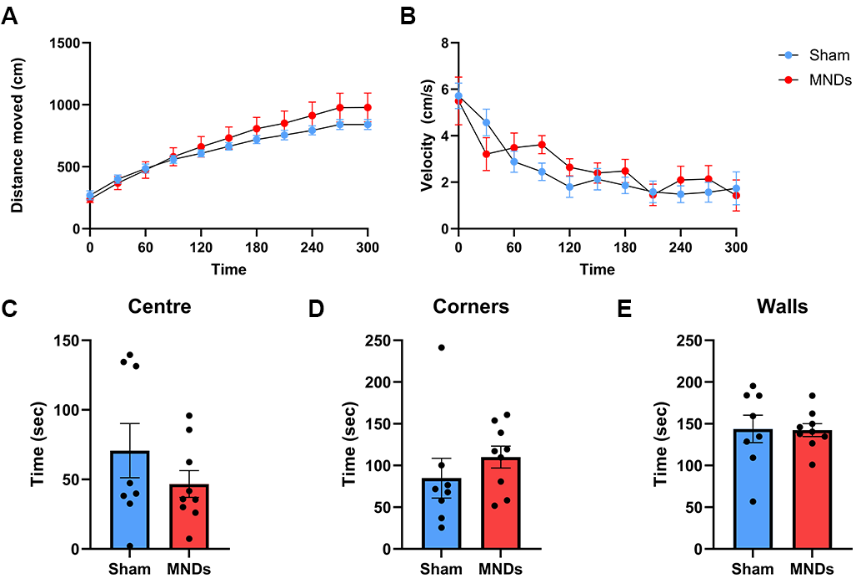
**Figure S7. The effects of magnetomechanical STN DBS during the Open Field Test (OFT). A.** Total distance moved (in cm) in the OFT ± S.E.M. for sham (n=7) and STN mDBS (n=9) mice. There was a non-significant increase between the groups when considering 30-second time bins. **B.** Velocity (in cm/sec) in the OFT ± S.E.M. for sham (n=7) and STN mDBS (n=9) mice. There was a non-significant increase between the groups when considering 30-second time bins. **C.** Total time spent in the centre. **D.** Total time spent in the corners. **E.** Total time spent at the walls during the 5-minute OFT. Data presented as mean ± S.E.M.


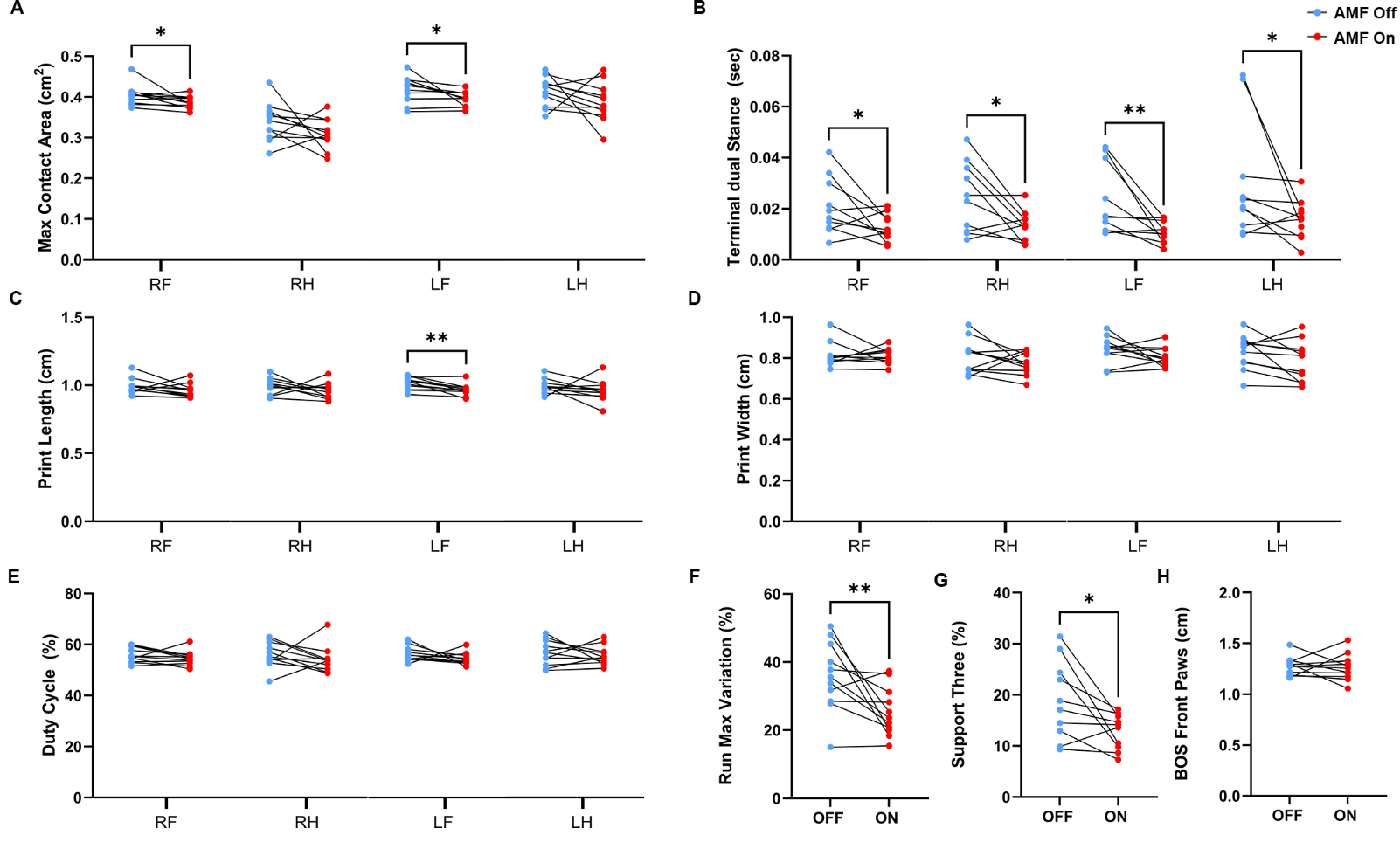
**Figure S8.** **Magnetomechanical STN DBS improves motor behaviour in naïve male mice.** Representative CatWalk XT results of four runs. AMF stimulation resulted in increased max contact area (p ≤ 0.0255) **(A)**, a decrease in terminal dual stance (p ≤ 0.0425) **(B)**, decreased print length (p = 0.0026) **(C)**, no change in print width (P ≤ 0.0358) **(D)**, no change in speed duty cycle **(E)**, a decrease in run max variation (p = 0.0075) **(F)**, a decrease in support three (p = 0.0134) **(G)**, and a no change in base of support front paws **(H)** between AMF On and AMF Off (n=11) as revealed by an independent one-tailed t-test. * Indicates p < 0.05, ** < 0.01. Data presented as mean ± S.E.M. RF: right front paw, RH: right hind paw, LF: left front paw, LH: left hind paw.

**
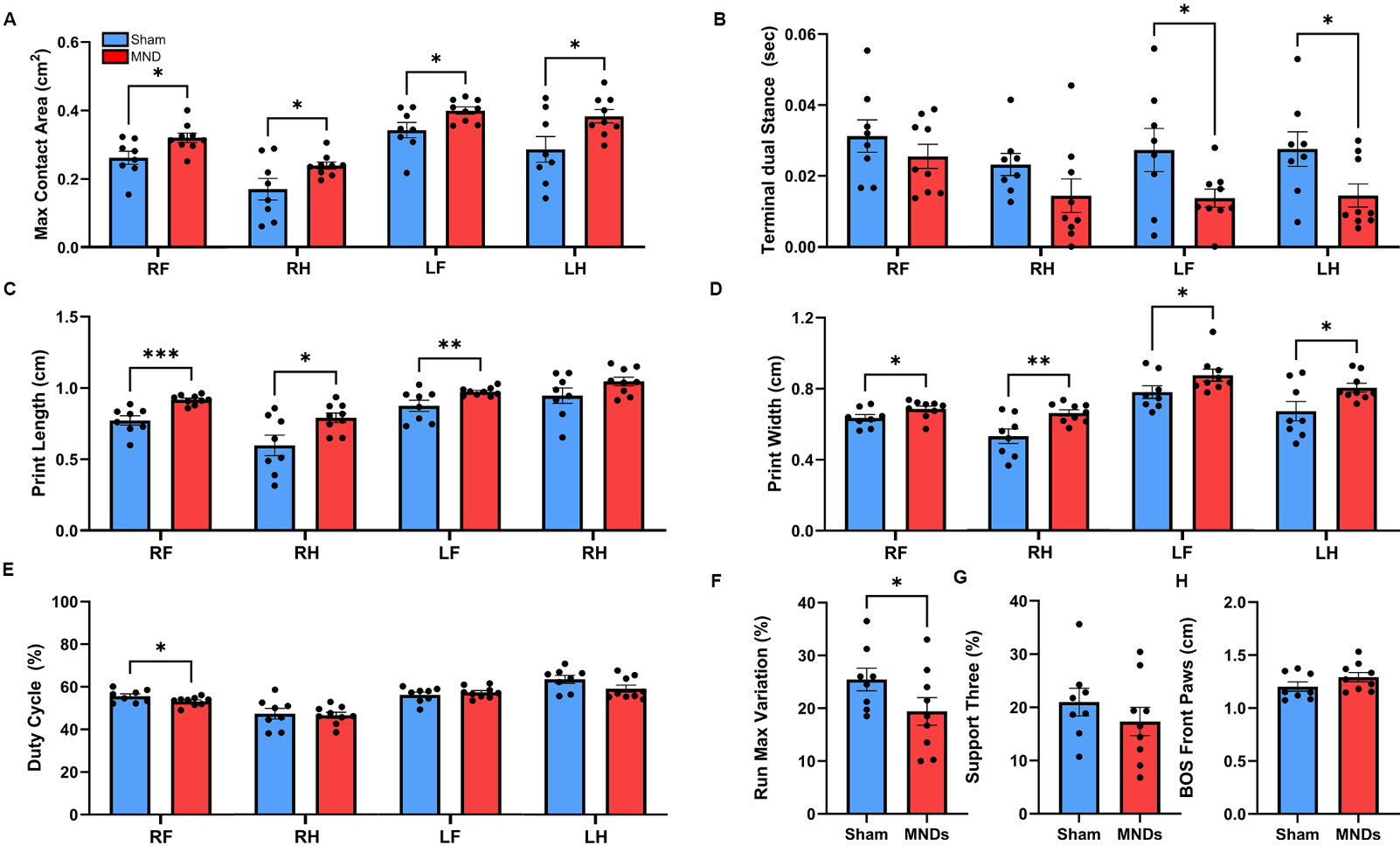
Figure S9. Magnetomechanical STN DBS alleviates severe Parkinsonian symptoms.** Representative CatWalk XT results of four runs. AMF stimulation resulted in increased max contact area (p≤0.0228) **(A)**, a left-sided decrease in terminal dual stance (p ≤ 0.0246) **(B)**, increased print length (p ≤ 0.0110) **(C)**, increased print width (P ≤ 0.0358) **(D)**, decreased speed duty cycle (p = 0.0352) **(E)**, decreased run max variation (p = 0.0485) **(F)**, no change in support three **(G)**, no change in base of support of front paws **(H)** between sham (n=9) and STN mDBS (n=8) as revealed by an independent one-tailed t-test. * Indicates p < 0.05, ** p < 0.01, *** p < 0.001. Data presented as mean ± S.E.M. RF: right front paw, RH: right hind paw, LF: left front paw, LH: left hind paw.

**
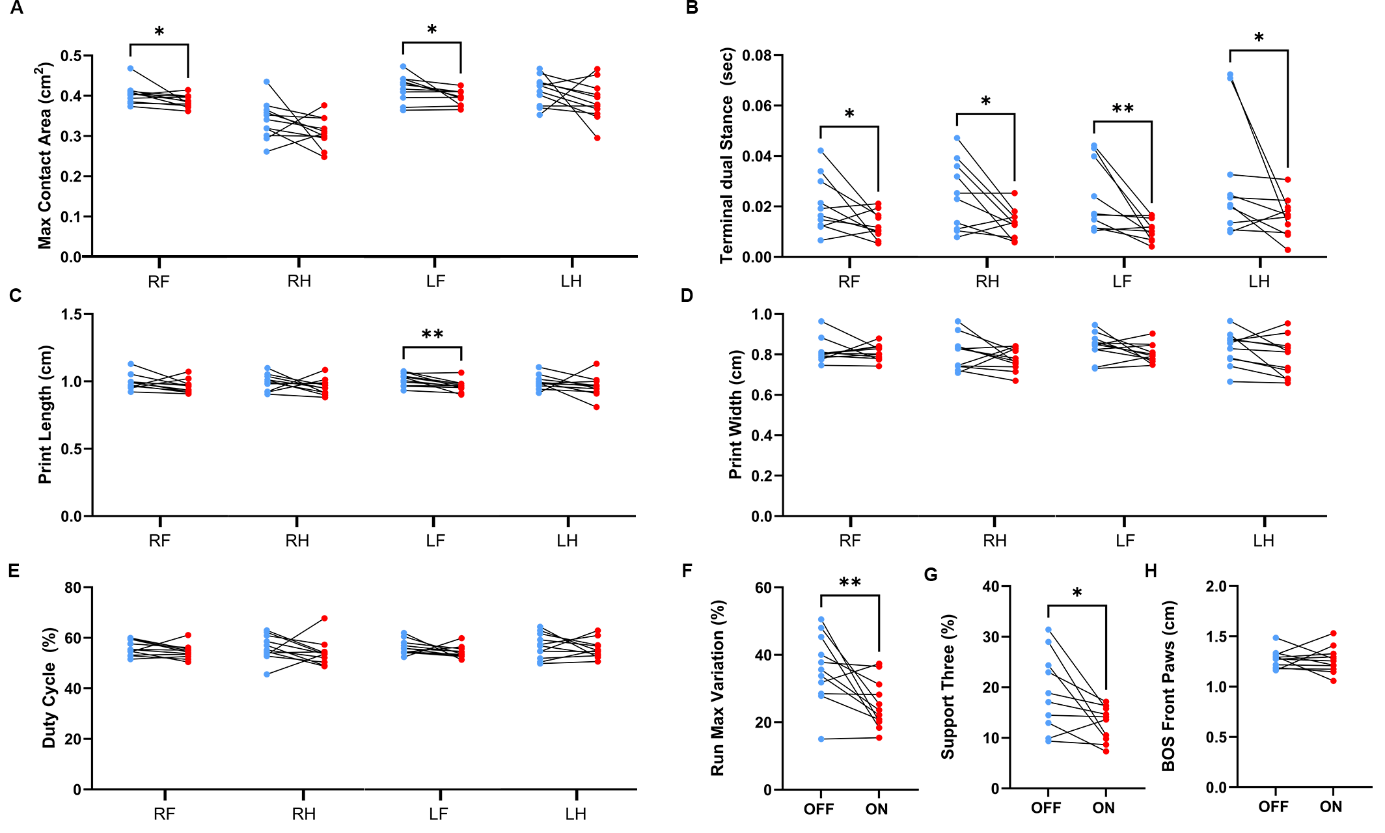
A B**


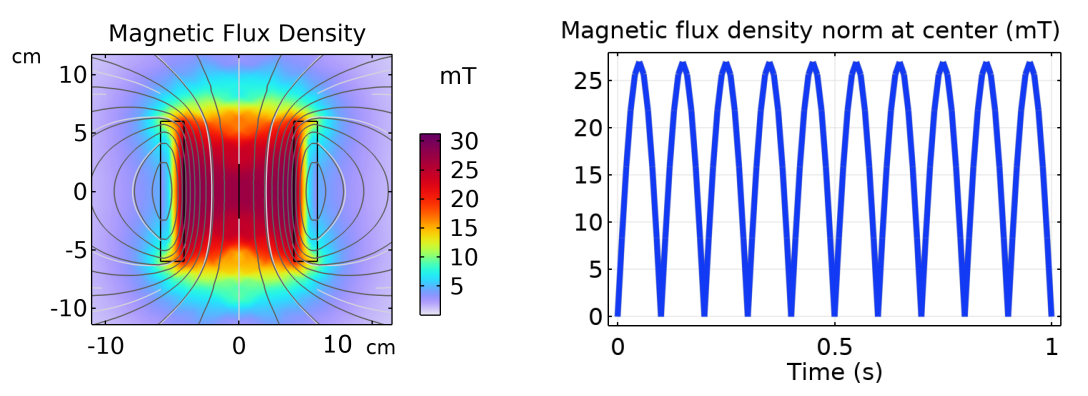


**Figure S10. Magnetic flux density produced by the coil during stimulation with a 5 Hz sinusoidal current of 3 A peak amplitude. A.** Spatial distribution of the magnetic flux density. **B.** Absolute magnetic flux density at the center of the field.
